# Emerging Cytokine Biosensors with Optical Detection Modalities and Nanomaterial-Enabled Signal Enhancement

**DOI:** 10.3390/s17020428

**Published:** 2017-02-22

**Authors:** Manpreet Singh, Johnson Truong, W. Brian Reeves, Jong-in Hahm

**Affiliations:** 1 Department of Chemistry, Georgetown University, 37th & O Sts. NW., Washington, DC 20057, USA; mks73@georgetown.edu (M.S.); jtt37@georgetown.edu (J.T.); 2 Department of Medicine, University of Texas Health Sciences Center at San Antonio, San Antonio, TX 78229, USA; ReevesW@UTHSCSA.edu

**Keywords:** cytokine sensor, optical protein sensor, nanobiosensor, ELISA, fluorescence, colorimetry, surface plasmon resonance, photonic crystal, optical waveguide, evanescent wave

## Abstract

Protein biomarkers, especially cytokines, play a pivotal role in the diagnosis and treatment of a wide spectrum of diseases. Therefore, a critical need for advanced cytokine sensors has been rapidly growing and will continue to expand to promote clinical testing, new biomarker development, and disease studies. In particular, sensors employing transduction principles of various optical modalities have emerged as the most common means of detection. In typical cytokine assays which are based on the binding affinities between the analytes of cytokines and their specific antibodies, optical schemes represent the most widely used mechanisms, with some serving as the gold standard against which all existing and new sensors are benchmarked. With recent advancements in nanoscience and nanotechnology, many of the recently emerging technologies for cytokine detection exploit various forms of nanomaterials for improved sensing capabilities. Nanomaterials have been demonstrated to exhibit exceptional optical properties unique to their reduced dimensionality. Novel sensing approaches based on the newly identified properties of nanomaterials have shown drastically improved performances in both the qualitative and quantitative analyses of cytokines. This article brings together the fundamentals in the literature that are central to different optical modalities developed for cytokine detection. Recent advancements in the applications of novel technologies are also discussed in terms of those that enable highly sensitive and multiplexed cytokine quantification spanning a wide dynamic range. For each highlighted optical technique, its current detection capabilities as well as associated challenges are discussed. Lastly, an outlook for nanomaterial-based cytokine sensors is provided from the perspective of optimizing the technologies for sensitivity and multiplexity as well as promoting widespread adaptations of the emerging optical techniques by lowering high thresholds currently present in the new approaches.

## 1. Introduction

Protein biomarkers and cytokines in particular play an increasing clinical role in early disease diagnosis, monitoring disease activity, and assessing therapeutic response. Hence, reliable quantification of cytokine levels, both in isolation and in combination, is extremely critical both in basic science laboratories and clinics. Advances in nanotechnology have led to solid-state biosensors featuring exquisite levels of detection sensitivity and much larger dynamic ranges than those provided by conventional platforms. In addition, such levels of detection can now be achieved with much reduced sample and reagent volumes and assay times as well as increased flexibility over the type and complexity of samples to be analyzed.

A number of detection principles exists which can be used for protein sensing, too numerous to be discussed in depth in this article. This review will focus on optical sensing methods which are the most commonly applied and serve as the gold standard. In addition, more recently developed, new optical platforms will be presented for cytokine detection. The specific optical principles covered in this Review are colorimetry, fluorescence, surface plasmon resonance, optical resonance, waveguiding, evanescence coupling, and interferometry. The fundamental core principles of each optical technique will be provided and specific examples of emerging technologies in cytokine detection will follow. In particular, this article focuses on recent sensor developments which capitalize on the unique optical properties of various nanomaterials, leading to vast improvements in overall sensor performance. Advantages and limitations of each optical technique in cytokine detection will be discussed while considering crucial sensor attributes such as sensitivity, selectivity, response time, sample requirements, multiplexing, throughput, cost, speed, and ease of use. For those new optical sensing paradigms, key challenges will be identified that need to be addressed before the sensors can be readily translated out of basic research settings into the clinical and healthcare domains. 

## 2. Cytokines: Biomarkers of Focus

Cytokines, chemokines, growth factors, and other proteins in physiological fluids and tissues can serve as potentially useful biomarkers [1,2,3,4,5,6]. In response to external pathogens, immune cells release low-molecular weight proteins such as cytokines and chemokines to regulate cell-to-cell communication, inflammatory responses, and tissue homeostasis. The host immune response involves a complex interplay of many biomolecules of which cytokines, chemokines, and growth factors are important players. Accordingly, these biomarkers are widely used to track and predict disease progressions as well as to monitor patient treatment outcomes [7,8,9]. The potentially decisive diagnostic importance of cytokine and chemokine biomarkers has continuously driven new research efforts to create novel sensors.

Cytokines are small, soluble proteins serving as signaling factors expressed by many different types of cells for immune regulation and response. Cytokines are both pleiotropic and redundant in that the same cytokine can exert multiple actions and different cytokines can signal similar responses, respectively [10]. This is due to the employment of the same receptor subunits and signaling pathways, such as for interleukin-4 (IL-4) and IL-13 [11]. These important biomolecules have been shown to be involved in many regulatory processes and immune responses to illnesses such as carcinomas [12,13,14], sepsis [15], inflammatory diseases [16,17,18], T-cell differentiation [19], and cardiovascular disorders [20,21]. When comparing patients with clinical disease symptoms to control subjects, the presence of various cytokines, chemokines, and growth factors can be either relatively repressed or elevated [22,23]. Further, during a systemic response to homeostatic disruption, the immune system can rapidly undergo a dynamic and complex transition from a pro- to anti-inflammatory response, making it important for clinicians to be able to rapidly and repeatedly assess dynamic changes in a patient’s status using multiple biomarkers for monitoring disease progression.

Chemokines, which are small chemotactic cytokines that influence cell migration and activation, are also key molecules in inflammation pathways and tumor regulation [24]. Chemokines also represent useful markers of various disease pathogeneses. Since they are generally present in higher amounts relative to other cytokines due to redundancies in their activation pathways and widespread production, chemokines have generally been easier to detect using traditional methods such as immunoassays and mass spectrometry [25].

Table 1 lists ~30 cytokines grouped into different categories which include cytokines, chemokines, growth factors, monokines, and others. The classification used in the table should be considered as more of a general guide instead of a definitive one, as some cytokines with multiple biological functions and activities can be placed in more than one class. For the purpose of this review, we will refer to all potential protein biomarkers catalogued in Table 1 as cytokines, regardless of their specific class.

## 3. Biosensors

### 3.1. General Biosensor Criteria

Key biosensor criteria include sensitivity (the response of a sensor per unit change in analyte concentration), selectivity (the ability for a sensor to discriminate between different analytes), dynamic range (i.e., linearity, concentration range over which the measured signal does not deviate from the ideal curve), response time (the time required for a sensor to indicate a defined percentage of its final response), accuracy (closeness of the sensor reading to the true value), precision (reproducibility of the sensor output), detection limit (DL, also known as limit of detection, the lowest concentration of analyte producing a measurable signal response), lifetime (the time period over which a sensor can be used without significant deterioration in performance characteristics), and stability (the change in the sensor’s baseline or sensitivity over a fixed period of time).

Of these criteria, the sensitivity and selectivity are the two major parameters under consideration for cytokine sensors used in a basic research laboratory setting. The sensitivity and selectivity of a sensor are related to its DL and specificity, respectively. For statistical and clinical validation of biomarker detection, the sensitivity and specificity of the biosensor should be determined in physiologically relevant contexts. In more applied sensor applications beyond the bench-top operation, factors such as multiplexing, throughput, coefficients of variance, response time, cost, and user-friendliness also become critical considerations. In addition, sensor configurations and detection approaches that can be facilely integrated with existing technologies and instrumentations are much sought after. Such integration capability is particularly warranted in high-throughput applications such as screening large libraries of protein and small molecule interactions as well as for screening multiple biomarkers or single biomarkers in a large number of samples. 

As protein immobilization strategies can influence sensor performance, identifying efficient coupling methods to link proteins on solid (sensor) surfaces is another crucial aspect in developing protein sensors and, therefore, it represents an active area of study. Overall, the coupling schemes that are commonly utilized are based on physical (electrostatic adsorption), chemical (covalent bond formation), and biological (high affinity biological moieties) approaches. Although these examples will not be discussed in detail in this paper, many review articles in the literature can provide a good overview of the different protein immobilization methods [26,27,28,29,30,31,32].

### 3.2. Specific Needs in Cytokine Sensors

Among those cytokines identified as potential biomarkers of various disease states, many important candidates are known to be present at very low levels. Hence, there is an increasing need to lower the DL of biomolecular assays to improve the clinical utility of the detection method. Typical concentrations of cytokine biomarkers in healthy individuals compared with those in various disease stages can span many orders of magnitude [33]. Accordingly, biosensors must also be able to quantitate over a great dynamic range of concentrations. The varying basal levels and disease-related profiles of different individual biomarkers create a strong need for advanced sensors that can enable dynamic measurements of a panel of biomolecules simultaneously and rapidly. In a clinical setting, monitoring relative changes of a panel of different biomarkers over several time-points to correlate with the disease phenotypes in a patient will be more reliable than single time-point analyses, since biomarker concentrations can vary over time depending on the degree of disease progression and treatment efficacy.

Developing quantitative sensors capable of measuring protein concentrations from different types of physiologically relevant samples is also crucial for biomarker detection. Cytokine concentrations have been shown to vary between various specimens sampled at the same time from blood, serum, plasma, urine, and saliva [22]. For example, TNF-α was found to be present in lower amount in saliva relative to plasma [14]. Therefore, the diagnostic utility of a particular cytokine as a biomarker may be promoted by having further information on the best physiological fluids showing the highest abundance of the cytokine of interest to be tested with a sensor. As these disease-relevant biomarkers are clinically identified in a wide array of physiological fluids, the sensor should be robust enough to withstand the potentially harsh biochemical environment of various types of patient samples while retaining sensor selectivity.

Physiological fluids, especially plasma and serum, contain protein abundances that can span dynamic ranges of over ten orders of magnitude [34]. Hence, the dynamic range of new sensors should demonstrate vastly improved response ranges than those achieved from conventional testbeds. At the same time, the basal levels of cytokines in healthy people are often well below the DL of standard assay kits, and many of the cytokine biomarker candidates cannot be successfully detected. For example, in an effort to evaluate the cytokines of β-NGF, TNF-β, IL-2, IL-15, and MIP-1α as potential biomarker candidates in diseases such as pelvic inflammatory disease, HIV infection, and tuberculosis, none of the biomarkers could be detected in any of the physiological samples of any patients examined [22]. Therefore, it is important to have a sensitive and selective enough biosensor that can quantify cytokines in complex matrices of physiological fluids over a wide concentration range. Hence, novel sensors developed for the quantitative measurements of cytokines in clinically relevant and biologically complex samples are currently faced with the simultaneous challenges of achieving much enhanced DLs and dynamic ranges relative to sensors that are presently available.

In addition to the need for high sensitivity, selectivity, and dynamic range, sensor platforms with multiplexing capabilities are highly valued. Many diseases involve complex and differential expression of multiple cytokines depending on the type and state of the disease. Accordingly, the use of multivariate biomarkers has been proposed to improve the prediction of survival for patients with cancer, autoimmune diseases, pathogenic infections, and cardiovascular disorders [35,36,37]. In those cases, evaluating single biomarkers was insufficient to confidently diagnose the conditions. Rather, multivariate biomarkers have been shown to be more effective than univariate biomarkers. For example, a panel of multiple biomarkers identified for familial Mediterranean fever has been shown to improve the accuracy and specificity of diagnosis relative to single cytokine measurements [38]. The need for comprehensive analyses of cytokine panels requires screening techniques capable of multiplexed evaluation of high numbers of samples rapidly. New sensors developed in array configurations may facilitate such high-throughput analysis.

To summarize, the ability to rapidly detect and quantify cytokines in clinically relevant concentrations will be critical in understanding the complex physiological role of the cytokines. It will also be of paramount importance in accurately assessing the potential of cytokines to serve as indicators for diagnosing and monitoring disease progression and evaluating therapeutic responses. Next-generation cytokine sensors should be capable of pushing the DL toward the attomolar (aM) level and providing dynamic ranges greater than the conventional 3–5 orders of magnitude. Along with the improved sensitivity and dynamic range, multiplexing and throughput capabilities need to be integrated to simultaneously measure more than several cytokines in a sample (or a single type of cytokine in several samples). Beyond bench-top applications, newly developed sensor platforms should offer robust, rapid, facile, and affordable detection strategies to aid in the seamless translation of the technology into clinical and point-of-care (POC) testing environments.

## 4. Sensor Improvements via Nanomaterials

The specific needs outlined in the previous section have led to the development of a wide array of sensors using different optical, electrical, electrochemical, acoustic, and hybrid transduction methods that often report DLs in the picomolar (pM) range. In particular, novel materials known as nanomaterials have been extensively explored in such development efforts due to their reduced size for sensor miniaturization as well as useful nanoscale properties for improved DLs. Nanomaterials can be synthesized in zero-dimensional (0D), 1D, and 2D forms whose shapes can vary from nanoparticles (NPs), nanotubes (NTs), nanowires (NWs), nanorods (NRs), and ultrathin films [39]. Table 2 displays the shapes and types of nanomaterials belonging to these different forms. As an example, the different nanomaterial forms consisting of the same chemical element of C, i.e., carbon allotropes, are shown in blue for the 0D (C bucky-ball), 1D (CNT), and 2D (graphene sheet) structures.

Recent advancements in the ability to synthesize and integrate these nanomaterials into biomarker sensing devices [40] have led to significant progress in pushing the current DLs for cytokines. For example, some recently demonstrated optical sensors have shown improved performance in cytokine detection with the use of gold nanorods (AuNRs) [41] and zinc oxide nanorods (ZnO NRs) [42,43]. Although not the topic of this review, the use of metal and metal oxide NPs [44,45,46], carbon nanotubes (CNTs) [47,48], and graphenes [49,50] in electrical and electrochemical devices have also led to improvements in surface acoustic wave (SAW)-based mass transducer sensors and amperometric- and field-effect-transistor (FET)-based sensor performance [51,52].

## 5. Cytokine Sensors with Various Optical Modes of Detection

Cytokine sensors based on optical modes of detection are the most widely used configurations in assays ranging from more traditional, qualitative assays performed in an aqueous phase to more recent, quantitative solid-state analyses performed in an array format. Depending on the specific optical sensors, labelled or label-free samples can induce changes in the absorbance, fluorescence, surface plasmon resonance, surface evanescence, optical resonance, and interferometric reflectance signal from the device to quantify the analyte of interest. Some of these optical techniques require the use of labels such as fluorophores (organic dyes, semiconductor nanocrystals, and quantum dots), organic and inorganic chemical reagents (reducing agents, colloidal metal catalysts), biological tags (enzymes with chromogens, native and modified green fluorescent proteins) and mass-increasing tags (high-mass molecules and compounds). More recently, label-free optical modes of detection have also been introduced. The ensuing section will discuss each optical detection mode in detail, summarizing the fundamental principles, measurement parameters, advantages and disadvantages, detection requirements, typical sensor capability, and demonstrated applications in cytokine detection. It is worthwhile to note that, although cytokines are selected as the protein of focus in this Review, all optical techniques discussed in this paper can be broadly applicable for any type of protein detection, not specifically limited to cytokines.

In addition to the DL, other essential sensor parameters such as the multiplexing and throughput capabilities, signal response time, total assay time, affordability, ease of use, and sensor compactness will be discussed. Additional criteria that will be specified for each novel sensing approach include, when appropriate, the sample preparation steps, the instrumentation needs, the ability to discern physiologically relevant concentrations, and the capacity to analyze complex biological fluids.

### 5.1. Colorimetry

Various colorimetric and turbidometric assays such as the Bradford assay, the Lowry assay, the biuret assay, and nephelometry are still in use and have advantages in the ease of performance and low-cost [53]. These approaches are liquid-phase assays that typically require large sample volumes (hundreds of μL or more) of samples and have limited sensitivity. In contrast, solid-phase colorimetric assays can be carried out on array or plate surfaces while using small amounts (a few μL or smaller) of assay agents. Solid-phase approaches also enable rapid and simultaneous assays involving a large number of samples. The colorimetric methods discussed in this section will be narrowed to the solid state form. 

In particular, this section focuses on enzyme-linked immunosorbent assay (ELISA)-based efforts as they represent the most commonly used scheme for cytokine detection [54]. ELISA technology is often considered to be the gold standard method for cytokine detection in basic science, industrial research, and clinical laboratories. Nowadays, solid state ELISA assay platforms are commercially available for many cytokines. To further increase the detection sensitivity of ELISA, attempts such as the use of nanomaterials as well as other detection modes have been made [55,56,57]. These efforts will be discussed in detail in the following section.

#### ELISA

**Figure 1:**
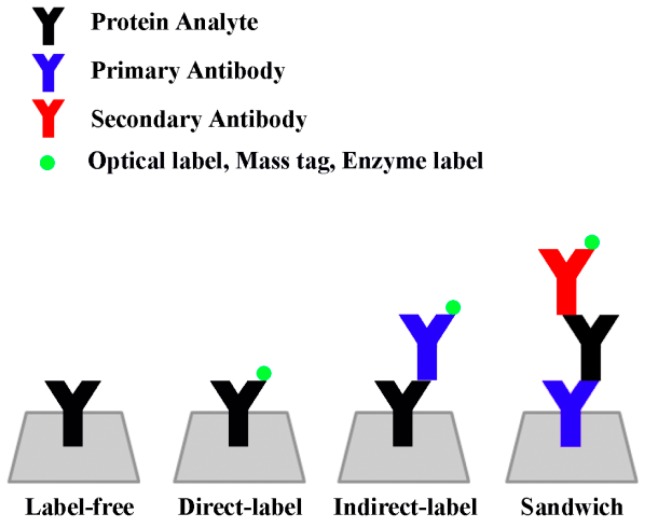
The different types of protein assay schemes used commonly in solid state sensor geometry. They are label-free (or direct), directly labelled, indirectly labelled, and sandwich type of assays. Labels targeting the analyte protein can be organic (fluorophores), inorganic (semiconducting nanoparticles, quantum dots, metallic nanoparticles), biological (enzymes), or mass-increasing compounds.

The primary detection mode of ELISA-based sensors relies on the colorimetric readings of chromogenic substances produced by the biochemical reaction of enzymes tagged to the biomarker proteins or their antibodies. The chemical product of this enzyme-substrate reaction can be easily read by a spectrophotometer or similar absorption scanner. In a typical “sandwich” ELISA detection scheme, capture antibodies are immobilized onto a surface that subsequently binds to antigens present in the sample. After this step, a second antigen-specific antibody coupled to an enzyme (detection antibody) is introduced and binds to the immobilized capture antibody/antigen complex. The ensuing addition of a chromogenic enzyme substrate generates a detectable optical signal. This method is generally considered highly selective for analytes, owing to the use of the two distinct epitope-binding antibodies designed to target specific analytes in the ‘sandwich’ assay scheme discussed above. Figure 1 shows the various types of assay schemes used for solid state protein detection. Frequently used pairs of enzyme-substrates include horse radish peroxidase (HRP)/o-phenylenediamine (ODP), HRP/3,3′,5,5′-tetramethylbenzidine (TMB), alkaline phosphatase (AP)/p-nitrophenylphosphate (p-NPP), β-galactosidase (β-gal)/o-nitrophenyl β-d-galactopyranoside (o-NPG), glucose oxidase (GOx)/β-d-glucose, and urease/urea.

In ELISA-based cytokine detection kits currently available commercially, the typical DL is in the range of several to tens pg/mL or higher. Various modification strategies have been used to achieve improved DLs. For one type of modified ELISA detection platform, a magnetic colorimetric assay was developed and employed for IL-6. The assay was carried out by immobilizing capture antibodies on magnetic Fe_3_O_4_ particles and labeling the secondary antibody with CeO_2_ spheres, a redox-active rare earth oxide material. The CeO_2_ label catalyzes the oxidation of *o*-phenylenediamine (OPD) to 2,3-diaminophenazine (oxOPD) whose process yields a yellow product with absorption at 448 nm. The DL was found to be 0.04 pg/mL and showed linearity between the range of 0.0001–10 ng/mL [58].

Attempts to improve the detection capability of the commercial ELISA kits were also carried out by making improvements in the detector, ELISA substrate, or detection mode itself. It is known that Au and Ag NP probes can be employed for metallic NP-enabled sensitivity enhancement [55,56,59,60]. In Ag enhancement, silver nanoparticles (AgNPs) linked to secondary antibodies are added in the immunoassay after which they act as catalysts to reduce Ag^+^ ions to Ag in the presence of a reducing agent such as hydroquinone. The process can enlarge the size of AgNPs up to 5 orders of magnitude and the deposited Ag amount can be quantified by colorimetry among other methods. Using this approach, a 15-plexed silver-enhanced sandwich immunoassay (SENSIA) was developed in a microarray configuration [61]. This effort made the colorimetry-based detection more affordable since the technique allowed signal detection for IL-10, IL-1β, IL-2, IL-4, IL-6 using a $100 flatbed scanner, an inexpensive alternative to array readers or laser scanners that are usually used for multiplexed cytokine monitoring with ELISA kits. A point-of-care ELISA test was also developed for IL-6 by using complementary metal-oxide-semiconductor (CMOS) sensors in standard smartphone cameras as a new detector to analyze the ELISA test. CMOS sensors convert photons into RGB pixels through filters composed of colored dyes that produce their own photoresponse. Although they are wavelength-independent detectors, wavelength-specific absorbance could be evaluated by distributing the spectrum of collected photons along one CMOS camera dimension. The use of the CMOS detector to perform the readout and quantification of an IL-6 ELISA assay enabled quantification over the same dynamic range of 2 to 125 pg/mL with a comparable DL to conventional detection equipment used in ELISA [62].

In other cases, the surface of the ELISA plates usually made out of glass, silicon, and plastic materials were modified with dendrimers. The dendrimer-coated ELISA plates were designed to provide increased sensitivity by reducing the nonspecific binding of proteins and antibodies by electrostatic interactions. The presence of the dendrimers also increased ligand capture efficiency by enabling favorable orientations of the capture antibodies. Subsequent detection of IL-6 in serum via luminol, IL-1β in serum via TMB, and TNF-α in buffer via TMB found DLs of 0.13, 1.15 and 0.48 pg/mL, respectively [63,64].

**Figure 2:**
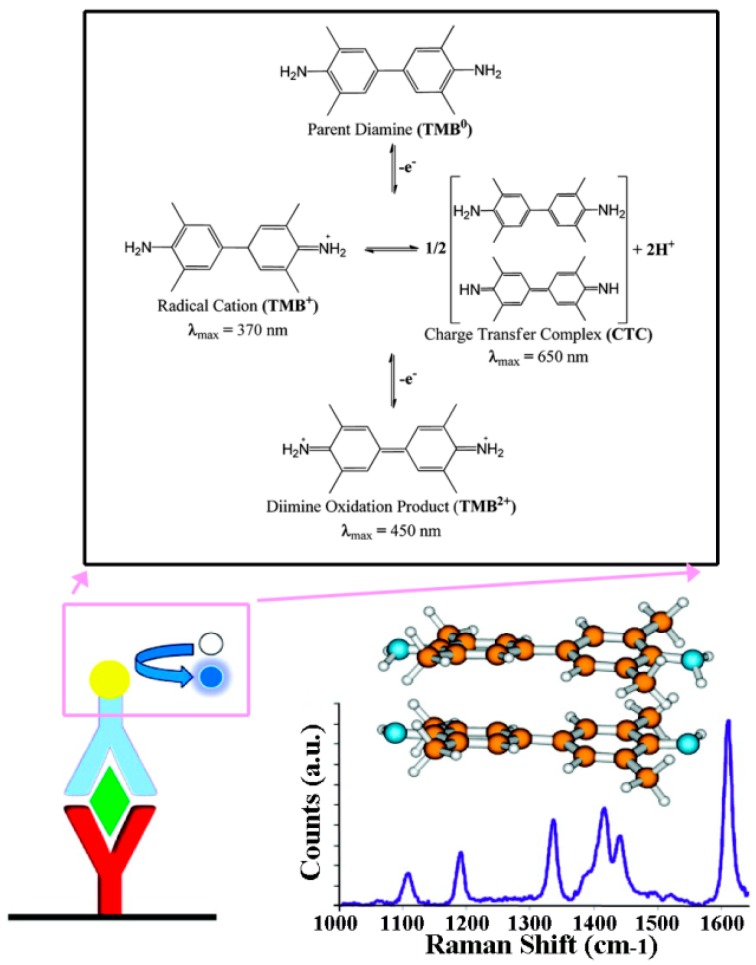
In a sandwich immunoassay involving HRP (yellow sphere)-labelled TNF-α antibody, the concentration of TNF-α analyte protein can be measured by examining the characteristic peaks for the oxidized products (indicated as blue sphere) of the TMB substrate (indicated as white sphere) whose conversion is triggered by HRP in the presence of H_2_O_2_. Reproduced with permission from Ref. [65] Copyright (2011) American Chemical Society.

In an approach using a commercial ELISA kit as-is, the conventional colorimetric detection of TMB was replaced with Resonance Raman (RR) spectroscopy [65]. To quantify TMB in solution and subsequently correlate it with TNF-α concentration, intensity changes in the RR spectra of the charge transfer complex, formed upon the HRP-catalyzed oxidation of TMB by H_2_O_2_, were recorded. The study showed that a 50 times lower DL could be achieved by changing the detection mode to RR from 4.50 pg/mL to 90 fg/mL for TNF-α in blood serum samples. The overall approach used and the typical RR spectra of the TMB products are displayed in Figure 2. Similarly, a traditional ELISA kit for IL-6 was employed in an amperometric detection scheme of HRP instead of colorimetric-based detection, which led to quantification within the improved dynamic range of 3.12 to 300 pg/mL [57].

### 5.2. Fluorescence

Protein detection based on fluorescence is especially popular and prevalent both in laboratory and clinical applications due to improved sensitivity and flexibility of the technique. When compared to a colorimetric, Bicinchoninic acid assay (BCA) for example, fluorescence-based protein assays have demonstrated important advantages such as rapid assay time (no need of long incubation steps), high stability (no time-dependent color development), better detection sensitivity, and simultaneous examination of multiple analytes [66]. With the continuing trend in sensor miniaturization and resultant reduction in sample volumes, there is an important challenge to push the DL below those of conventional fluorescence sensors. In many clinically relevant applications, the need to quantitate low-expressed cytokines demands even higher detection sensitivity, down to the concentration ranges of sub-fg/mL. Recent examples of nanomaterial-based approaches have proven to be highly promising in this respect, showing sufficiently high fluorescence sensitivity adequate for quantitative biomarker detection. 

In regards to multiplexing, wide varieties of fluorophores are available with characteristic absorption and emission peaks, which can be used as independent tags to examine multiple analytes simultaneously. As for detection flexibility, a wide range of commonplace instruments such as a fluorescence microscope, spectrophotometer, plate reader, array scanner, and flow cytometer can be used in collecting fluorescence signals. Along with improved sensitivity, these aspects of versatility and multiplexity in fluorescence-based approaches become crucial to protein biomarker studies.

#### 5.2.1. Fluorescently Encoded Microbeads

Fluorescence-based multiplexing capability can be best seen in commercially available cytokine readers using optically encoded microbeads. Fluorescent moieties of different wavelengths and levels of intensity are encoded internally by entrapment within micrometer sized-bead matrices or externally by covalent conjugation to the surface of the microbead. The microspheres can then either be suspended in microwells [67] or deposited onto different surfaces such as etched optical fibers in an array configuration for multiplexing [17]. In one example of such multiplexed cytokine detection, polymeric beads of 3.1 μm in size were first fluorescently encoded internally and dispersed into microwells formed within etched optical fibers. The microbead sensor was subsequently used for the parallel detection of ten different cytokines of VEGF, IP-10, IL-8, MCP-1, CCL2, TIMP-1, RANTES, MIP-1β, Eotaxin-2, and IL-6. Although the determined DLs were considerably higher than the comparative ELISA measurements, ranging from 8 to 469 pM (i.e., ~0.15–6.9 ng/mL for a ~15 kDa cytokine), this method was successfully used to assay the saliva supernatants of patients with pulmonary inflammatory diseases using 100 μL of sample and a total assay time of 2.5 h. Another study using multiplexed bead-based kits attempted to analyze the concentrations of 48 cytokines in the plasma, saliva, and urine obtained from twenty healthy subjects. Out of the 48 total cytokines, only 19, 14, and 4 cytokines could be detected from the plasma, saliva, and urine, respectively [22], underscoring the need to push the DL to a level that is relevant to the baseline concentrations of the cytokines and to access a wider dynamic range associated with different physiological fluids. 

In addition to the above-stated improvements of fluorescence techniques, further efforts towards even greater sensitivity and throughput have also been made. In this regard, fluorescence-based protein microarray assays have been developed atop a range of substrates including nitrocellulose, glass slides, aldehyde-modified glass, epoxy coated glass, and bovine serum albumin-N-hydroxysuccinimide (BSA-NHS) coated glass. In microarrays, the signal consistency and detection sensitivity are known to be largely influenced by the substrate surface. An ideal substrate would present zero background signal and uniform signal distribution on the reaction spots of the bioassays with the signal intensity being proportional to the target concentration. Hence, new substrate materials for microarrays are being sought to overcome the issues of nonspecific protein adsorption and high background to signal ratio. For example, a type of Teflon derivative, a fluorinated ethylene propylene (FEP) membrane, was recently developed as a fluorescence microarray substrate to provide an ultralow background signal in cytokine detection. Atop the FEP membrane, a polydopamine microspot array was fabricated for protein conjugation and this scheme yielded DLs for IL-1β, IL-6, and IL-10 of 8.91, 1.33, and 6.12 pg/mL, respectively [68].

Burgeoning research efforts in solid-state protein detection [35,69] have made possible the development of commercially available antibody arrays which now exist in several configurations including solid-state planar microarrays, semi-quantitative planar membrane arrays, and liquid-state microsphere-based suspension arrays. Additional coupling of antibody arrays with encoded microbeads has also been reported for further miniaturization of the spot footprint on the surface by 3–4 orders of magnitude [70]. The antibody-conjugated microbeads were created in 1 μm^2^ spot areas, greatly reduced from 2500 to 10,000 μm^2^ spots produced by conventional robotic spotting/printing methods. The arrayed sensor formats can be easily coupled with conventional fluorescence microscopy as well as flow cytometry to allow for discrimination of fluorescence emissions and intensities for analyte identification and quantification [17].

#### 5.2.2. Metal Enhanced Fluorescence

The presence of metals at an optimal distance away from a fluorophore-coupled analyte can significantly enhance the DL of the fluorescence-based sensors via a process known as metal enhanced fluorescence (MEF) [71,72,73,74]. The same process is also referred to as surface-enhanced fluorescence (SEF) and metal-induced fluorescence enhancement (MIFE) in the literature. In addition to MEF, there is a related metal-based scheme which modulates the surface plasmons and localized surface plasmons of metals such as Au and Ag to achieve fluorescence enhancement. This section will focus on describing MEF, whereas surface plasmon and localized surface plasmon based detection schemes will be discussed in detail in Section 5.3.

##### Fundamental Background for MEF

**Figure 3:**
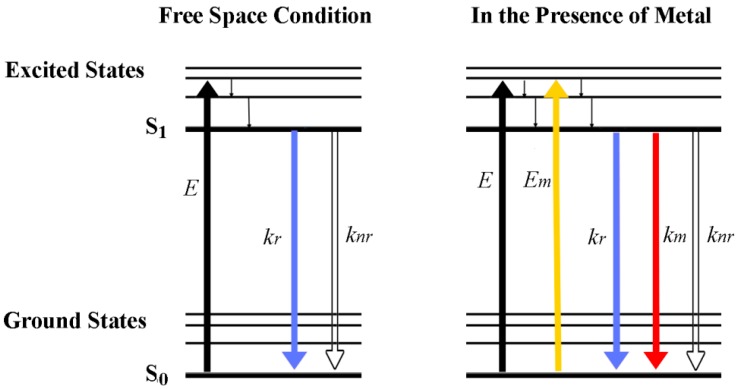
Schematics illustrating the excitation and emission processes of a fluorophore in the absence and vicinity of a metal. S_0_ and S_1_ represent the ground and excited electronic energy states, respectively. *E* denotes for the excitation energy whereas *k_r_*, *k_nr_*, and *k_m_* are rate constants associated with the different radiative and nonradiative emission processes.

In MEF, the concentrated electromagnetic field built around a metal is thought to increase the excitation of a fluorophore molecule from the original free space condition of *E* to *E* + *E_m_*, as depicted in Figure 3. In addition, during the emission of the fluorophore, an additional radiative decay process is considered in the presence of a metal positioned at an optimal distance from the fluorophore. The promoted interaction of the fluorophore with the metal is thought to increase the radiative decay rate of the fluorophore by *k_m_* from its intrinsic radiative decay rate of *k_r_* in the absence of a metal. As the radiative rate increases, the amount of emitted photons increases over time and the lifetime of the fluorophore decreases. The equation for the quantum yield, *Q*, of the fluorophore and its lifetime, *τ*, in the absence of the metal can be written as:

(1) $$Q=\frac{k_{r}}{k_{r}+k_{nr}+k_{q}}$$

(2) $$\tau =\frac{1}{k_{r}+k_{nr}+k_{q}}$$

with *k_r_*, *k_nr_*, and *k_q_* denoting for the rate constant of radiative decay, nonradiative decay, and quenching processes, respectively. 

In the presence of a metal, these expressions change to:

(3) $$Q_{m}=\frac{k_{r}+k_{m}}{k_{r}+k_{nr}+k_{q}+k_{m}}$$

(4) $$\tau_{m}=\frac{1}{k_{r}+k_{nr}+k_{q\;}+k_{m}}$$

where *k_m_* is the rate constant of a radiative decay process made available by altering the fluorophore’s free space condition with the optimally positioned metal. The presence of the metal does not significantly alter the *k_r_* in the equations as *k_r_* is defined by the oscillation strength of the fluorophore between the excited electronic state of S_1_ to the ground electronic state of S_0_. On the other hand, the effect of the metal is thought to decrease *k_nr_* and *k_q_*. The combined MEF scenarios for the excitation and emission of a fluorophore can lead to a stronger fluorescence intensity under optimized experimental conditions.

In particular, if the size of the metal is on the nanometer scale, the surface plasmons of the metal nanoparticles known as the localized surface plasmons, can collectively provide an exceptionally strong and highly localized electromagnetic field to the proximal fluorophore. The detailed mechanisms of surface plasmon and localized surface plasmon can be found in Section 5.3. In addition, the optical absorption spectra of metal NPs are strongly dependent on their shape. Metals of anisotropic shapes are expected to result in better excitation of the fluorophore since the electromagnetic fields around anisotropic objects are stronger compared to isotropic counterparts due to a phenomenon known as the lightning rod effect. This effect is associated with the metal’s surface plasmon resonance modes existing in the directions parallel and perpendicular to the long axis of anisotropic metal NPs (the longitunidal and transverse mode, respectively). In addition to MEF being affected by the surface plasmon strength of the metal, the extent of MEF can also be dependent on the wavelength of the incident light, the angle of illumination, and other factors. In fact, it is known that several variables, such as the distance between metal and the fluorophore and the overlap/offset degree in the absorption/emission spectra between the metal and the fluorophore, need to be considered to achieve the strongest MEF.

**Figure 4:**
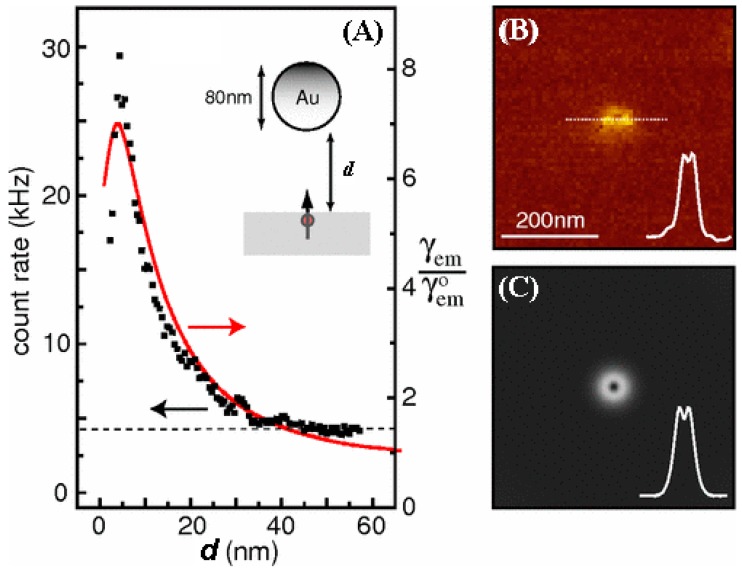
(**A**) The distance dependence of a fluorophore in the vicinity of a metal nanoparticle on the resulting fluorescence rate is shown. The solid and dotted data are from theoretical and experimental outcomes, respectively; (**B**) The fluorescence image is acquired from a single fluorophore molecule located at 2 nm away from the metal. The dip in the center is due to fluorescence quenching; (**C**) Corresponding theoretical image for (**B**) is provided. Reproduced with permission from Ref. [76] Copyright (2006) American Physical Society.

One caveat is that the fluorophore should be placed at an optimal distance away from the metal film or metal nanoparticle surface for the fluorescence enhancement effect to occur [75]. This is due to the fact that the interaction between the fluorophore and the metal is largely governed by two competing pathways [76]. One is the strong local field increase in the fluorophore’s dipole moment near the metal’s surface plasmon resonance which promotes the radiative decay rate. The other is the non-radiative energy transfer from the excited fluorophore to optically inactive electronic excitations in the metal. This interplay between the strength of the electromagnetic field and the non-radiative energy transfer is strongly affected by the separation distance, *d*, of the fluorophore from the metal surface. Therefore, fluorescence signal can be enhanced only at some optimal distance range of *d*. If the two are too closely placed, fluorescence emission is quenched due to the suppression of the quantum efficiency by the dominant non-radiative process. If they are too far apart, the degree of enhanced electromagnetic field built around the metal or the energy transfer between the metal and fluorophore becomes too weak to lead to an increase in fluorescence intensity. This relationship is known to follow either a ~1/*d*^6^ or ~1/*d*^4^ dependence for a system involving a Forster resonance energy transfer (FRET) or surface energy transfer (SET) process, respectively [75,77]. Due to these reasons, both enhancement and quenching have been widely observed in fluorescence experiments in the literature interfaced with metals of Au and Ag. Similar distance-dependent phenomena are also observed in surface plasmon resonance-based sensors which are discussed in Section 5.3. For fluorescence enhancement, the optimal distance of the fluorophore to the metal surface can be approximated to be between 5 and 20 nm [78], as shown in Figure 4.

##### MEF Applications in Cytokine Detection

**Figure 5:**
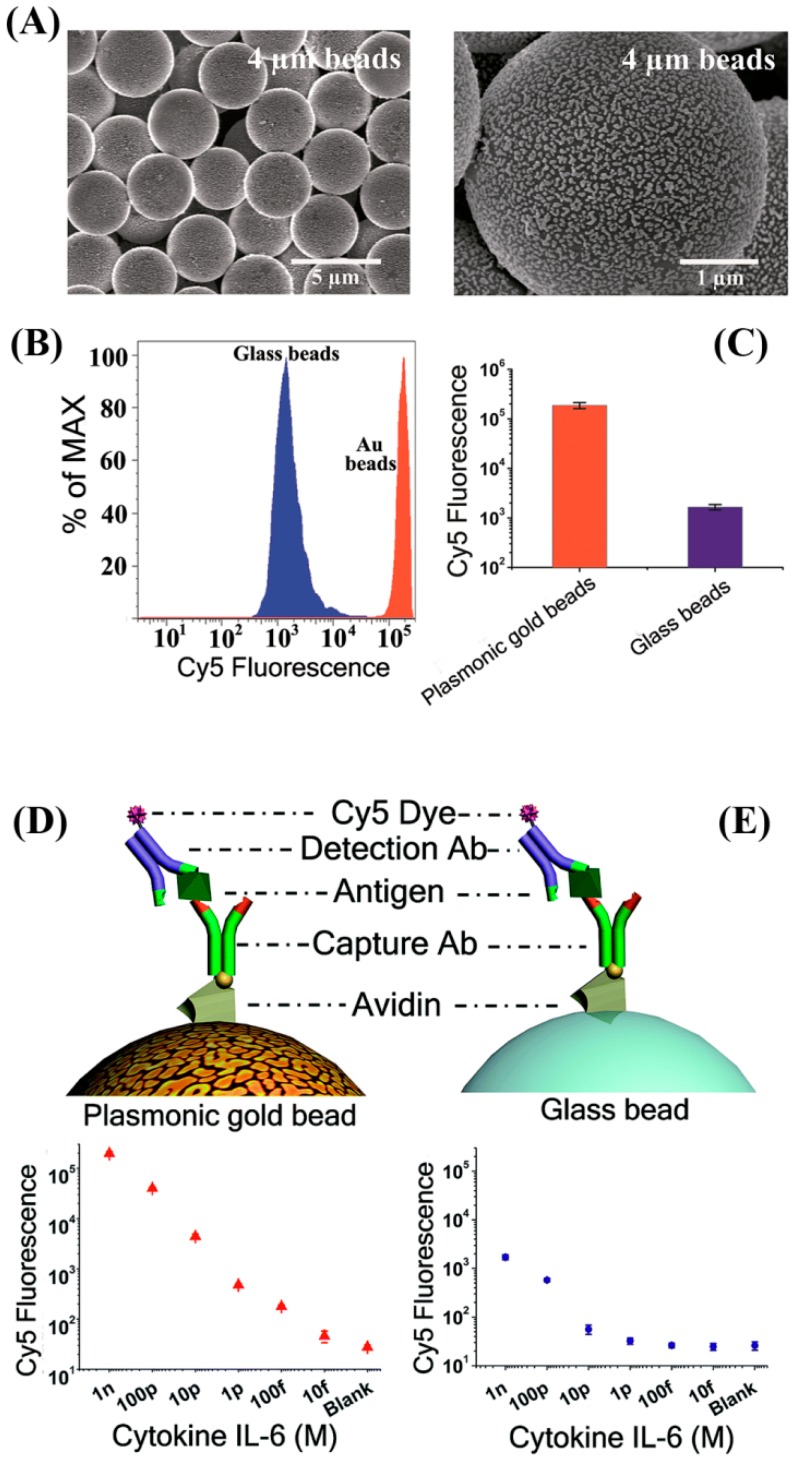
(**A**) Scanning electron microscope images of plasmonic Au nanoisland-covered glass beads are displayed; (**B**) Comparison of the fluorescence signal from the Cy5-avidin coated plasmonic gold beads versus glass beads in flow cytometry; (**C**) The mean Cy5 fluorescence intensity plot shows an enhancement of ~2 orders of magnitude on the plasmonic bead relative to the glass bead; (**D**) Sandwich assay schemes for IL-6 detection on a plasmonic Au bead and on a glass bead are shown; (**E**) Fluorescence data for IL-6 detection on (left) plasmonic gold beads and (right) glass beads are plotted as a function of the protein concentration. Reproduced with permission from Ref. [79] Copyright (2014) Royal Society of Chemistry.

The fluorescence enhancement phenomenon in the presence of noble metals has been exploited in cytokine detection. An enhanced sensitivity up to two orders of magnitude was realized in the multiplexed fluorescence detection of IL-6, IFN-γ, IL-1β, and VEGF by coating the 4–8 μm glass microbeads with nanosized Au islands (~100 nm in size with 10–30 nm spacing), as shown in Figure 5A [79]. When using Cy5 as a reporter molecule, the lowest reported DL was 0.2 pg/mL for IL-6, whose value is similar to a previous work which used a planar substrate of a Au film-coated glass slide in multiplexed cytokine detection [80]. Figure 5 displays the Cy5 fluorescence signal enhancement observed using flow cytometry while employing the plasmonic gold beads as well as the conventional glass beads. Figure 5 also shows the fluorescence responses obtained from the two bead types as a function of the IL-6 concentration. In another study comparing the use of plasmonic Au chips versus conventional microarrays, an improved detection down to a 0.2 pg/mL DL was similarly achieved for another cytokine of IL-20 in serum and synovial fluid [81].

#### 5.2.3. Zinc Oxide NR Enhanced Fluorescence

##### Fundamental Background for Enhancement enabled by Zinc Oxide NRs

The bases of fluorescence enhancement demonstrated in optical biodetection using ZnO NRs can be considered both from the excitation and emission processes. During fluorophore excitation, the highly anisotropic geometry of ZnO NRs can result in much stronger electromagnetic fields around the NRs compared to their isotropic counterparts such as ZnO thin films through a similar dimensionality and shape effect that was discussed earlier. This, in turn, can lead to better excitation of the fluorophores, producing higher fluorescence signals. For the emitted signals, the NR forms of ZnO are known to serve as an exceptional light coupling and light guiding medium, particularly as a subwavelength waveguiding medium. In this regard, the detailed mechanisms for the enhancement related to emission are discussed in Section 5.5 and Section 5.6, pertaining to subwavelength waveguiding and surface evanescent wave propagation, respectively.

##### Biomolecular Fluorescence Enhancement via Zinc Oxide NRs

**Figure 6:**
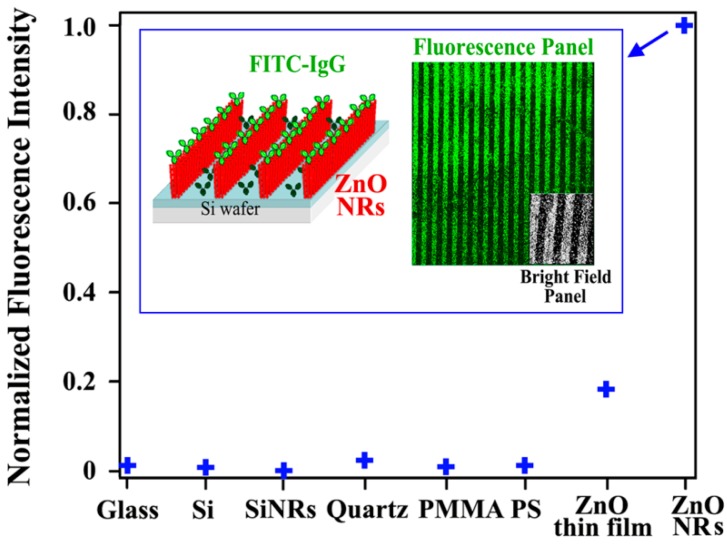
Normalized fluorescence signals from fluorophore-linked immunoglobulin G (IgG) deposited on various platforms are compared. In this comparison experiment, protein deposition conditions were kept identical for all cases. Biomolecular fluorescence emission on ZnO NRs platform was the highest when compared to the emission intensities measured on conventional substrates such as glass, silica, quartz, and polymers. Adapted with permission from Ref. [83] Copyright (2016) American Chemical Society.

Biomolecular fluorescence emission profiles have shown to be significantly increased on ZnO NRs relative to the same biomolecule’s emission on other control surfaces [82,83,84]. This effect is displayed in Figure 6 comparing the fluorescence intensity of a model fluorophore-coupled protein on ZnO NRs versus other substrates such as glass, quartz, polystyrene (PS), polymethylmethacrylate (PMMA), and Si with a native oxide layer. To assess the effect of chemical composition and dimensionality of the materials on the biomolecular fluorescence intensity, additional controls such as ZnO thin films (a two-dimensional (2D) counterpart to the 1D ZnO NRs) and SiNRs (a 1D material of comparable sizes to the ZnO NRs but made from Si) were also examined. In all comparisons, the ZnO NRs exhibited extremely stronger biomolecular fluorescence relative to the intensities from the controls and conventional platforms.

The biomolecular fluorescence enhancement is influenced by the dimensionality of ZnO as evidenced by the biomolecular fluorescence on a 2D ZnO thin film, which was enhanced relative to the conventional materials but showed only ~20% of the signal increase observed from the ZnO NRs. At the same time, it was also determined that the biomolecular fluorescence enhancement is unique to the material of ZnO and is not due to the inherently high surface-to-volume ratio of the 1D nanomaterials contributing to greater biomolecular adsorption leading to an increase in the fluorescence signal. Even for cases in which there were up to three orders of magnitude higher biomolecular concentrations on the conventional platforms relative to the deposited amount on ZnO NRs, the biomolecular fluorescence signal on ZnO NR platforms was still much stronger than those on the control surfaces. Other nanomaterials with similar or smaller sizes (i.e., comparable or higher surface-to-volume ratio) relative to the ZnO NRs led to a negligible signal increase or even fluorescence quenching. ZnO NRs-enabled fluorescence enhancement is achieved without any quenching even when fluorophores are placed directly on top of the NR unlike the MEF case. ZnO NRs are optically transparent in the visible and near infrared region and, hence, they show no spectroscopic overlap with the fluorophores commonly used in bioassays.

**Figure 7:**
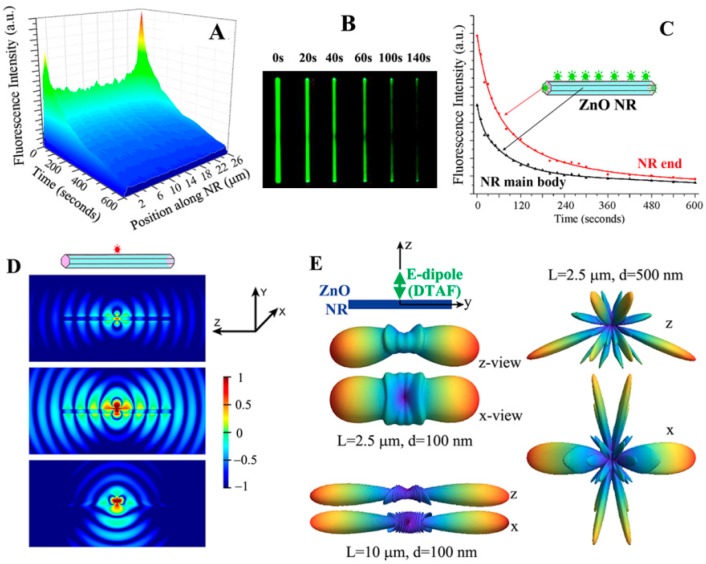
Spatially localized, temporally extended biomolecular fluorescence signal observed on individual ZnO NRs. (**A**,**B**) The contour map in (**A**) and the time-lapse images in (**B**) display the fluorescence intensities of fluorophore-linked IgG antibodies, DTAF-antiIgG, measured along the long axis of a 25 μm-long ZnO NR; (**C**) Differences in the time-dependent decay profile of the fluorescence intensity were clearly observed depending on the ZnO NR crystal facets. Red and black data show biomolecular fluorescence measured from the NR end and main body, respectively; (**D**) FDTD simulations were carried out to obtain the radiation patterns from a single emitter radiating at 576 nm placed 10 nm from the ZnO NR surface, with the dipole oscillation polarized along the *X*-(top), *Y*-(middle), and *Z*-(bottom) direction; (**E**) The dimensional effect of ZnO NRs on *FINE* was evaluated by simulating far-field radiation patterns of a 517 nm electric dipole. A pair of far-field patterns is shown for each NR of the specified length (L) and width (d) where the top/bottom simulation corresponds to the spatial patterns observed from the *Z*-/*X*-axis. Reproduced with permission from Ref. [85] Copyright (2014) Royal Society of Chemistry.

**Figure 8:**
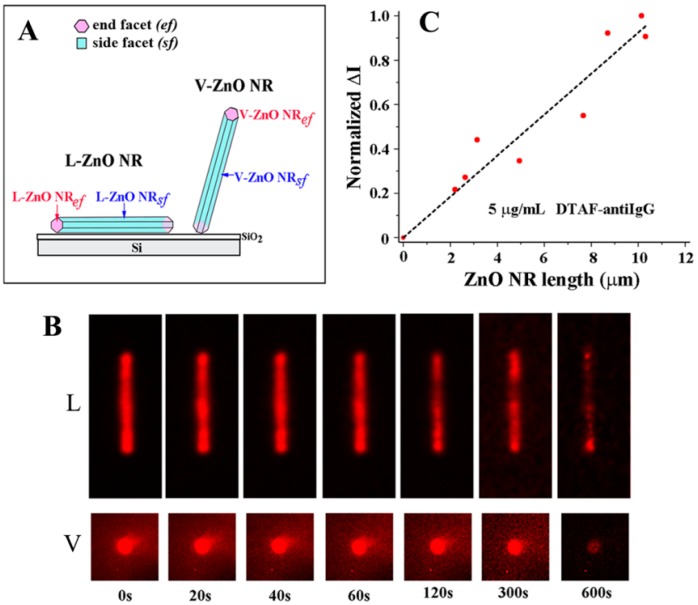
(**A**) The different growth orientations of the ZnO NRs are schematically shown; (**B**) The presence of *FINE* was confirmed for all cases when the spatial and temporal emission behavior of fluorophore-linked IgG antibodies, 1 μg/mL TRITC-antiIgG, was monitored in time-lapse fluorescence panels, as shown for the case of a L-ZnO NR (top) and a V-ZnO NR*_ef_* (bottom). The magnitude and degree of *FINE* were much higher for V-ZnO NRs relative to L-ZnO NRs; (**C**) ZnO NRs of various lengths and widths were analyzed after treating them with IgG antibodies coupled to a different fluorophore, 5 μg/mL of DTAF-antiIgG. In all cases, the difference in the normalized fluorescence intensity measured at the NR end versus NR side facets, Δ*I* = *I*_avg,NR*ef*_ − *I*_avg,NR*sf*_, indicated that the degree of *FINE* increased as the NR length became longer. The NR width did not lead to a significant effect. Reproduced with permission from Ref. [86] Copyright (2015) Royal Society of Chemistry.

A unique phenomenon of fluorescence intensification on NR ends (*FINE*) was discovered upon examining biomolecular fluorescence intensities along the different positions on single ZnO NRs. Unlike the spatially uniform signal observed on conventional polymeric substrates, both the biomolecular fluorescence intensity and photostability were not only much increased relative to those on polymers but also highly intensified at the NR ends, as seen in Figure 7. This effect was corroborated by finite difference time domain (FDTD) computer simulations. The simulations showed that the emission from the electric dipole, i.e., fluorophore, is effectively guided along the NR surface as evanescent waves as well as inside the NR after coupling into the ZnO NR subwavelength-waveguiding media which, finally, radiates out to the far field from the two NR ends. The degree of *FINE* was dependent on the physical sizes and spatial orientations of the ZnO NRs, increasing as the NR length becomes longer and as the NR orientation is close to vertical (V-ZnO NR) rather than lateral (L-ZnO NR) with respect to the underlying growth substrate. Data shown in Figure 7 and Figure 8 summarize the key findings of *FINE* on ZnO NRs and the associated nanomaterial factors influencing *FINE*.

##### ZnO NR Applications in Cytokine Detection

**Figure 9:**
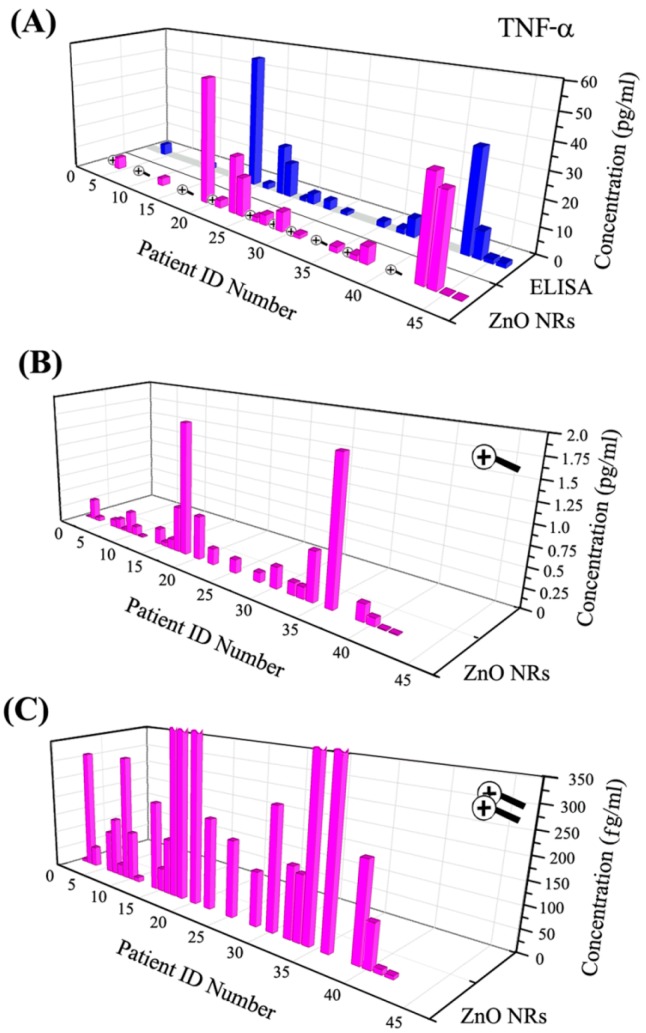
(**A**) The 3D bar graphs display the TNF-α concentrations in ICU patient urine samples measured by the ELISA- and ZnO NRs-based platforms for comparison. The grey regions in the ELISA row correspond to missing concentration data, indicating that the TNF-α levels in the samples were below the ELISA DL of 5.5 pg/mL. In contrast, ZnO NRs were able to measure the TNF-α concentrations of all 46 patients. The magnifier signs inserted in the ZnO NRs row correspond to the patients that belong to the grey area of the ELISA-based assay, and the bar graphs of these patients are shown separately in (**B**,**C**) for clarity; (**B**,**C**) The zoomed-in 3D bar graphs are the missing TNF-α concentrations that were revealed by the ZnO NRs-based assay. The upper limits of the vertical ranges in (**B**,**C**) are adjusted to 2 pg/mL and 350 fg/mL, respectively, in order to show the variations in the TNF-α concentrations between patients more clearly. The truncated bars indicate that their TNF-α concentrations exceed the upper limit of the 3D graph. Reproduced with permission from Ref. [87] Copyright (2016) Royal Society of Chemistry.

ZnO NRs have been extensively evaluated for their use as an optical platform to permit enhanced fluorescence detection of DNA and proteins both in simple [82,83,84] and more complex assay environments [42,87,88]. The most recent development in the area of ultrasensitive fluorescence detection using ZnO NRs pertains to the multiplexed detection and quantification of two urinary biomarkers, TNF-α and IL-8, in patients at risk for acute kidney injury (AKI) [87]. In an attempt to quantitatively compare the levels of TNF-α and IL-8 obtained via ZnO NRs-based assays to those attained using commercial ELISA-based detection platforms, the biomarker readings from the same patient samples were directly compared between the two methods. The DLs were determined as 4.2 (TNF-α) and 5.5 (IL-8) fg/mL for the ZnO NRs sensor and 5.5 (TNF-α) and 7.5 (IL-8) pg/mL for the ELISA-based kit. Figure 9 displays TNF-α assay results carried out with urine samples from patients in a medical intensive care unit (MICU). TNF-α levels from the same samples were measured with ZnO NRs-based assay and a commercial ELISA kit. ZnO NRs showed ultratrace levels of TNF-α concentration that were well below the DL of ELISA, as shown in Figure 9.

The ZnO NRs-based approach can provide direct advantages including facile platform fabrication, desirable optical properties, biocompatibility, and high multiplexing/throughput capacities. The ZnO NR arrays are easily fabricated using a gas-phase method through well-established synthesis procedures and can be used directly after growth without any post-synthetic modifications. Further, the highly crystalline NRs exhibit many desirable optical properties including no intrinsic fluorescence (i.e., absence of autofluorescence) as well as enhancement of the optical intensity and photostability of nearby signal emitters. Since the ZnO NRs do not display any photoluminescence in the visible and near-infrared range, they do not interfere with the spectroscopic profiles of fluorophores commonly used in bioassays. At the same time, the reduced dimensions and high shape anisotropy of the ZnO NRs enable optical enhancement and prolonged stability of the signal from fluorophore-tagged biomolecules adsorbed on their surface, allowing for the ultrasensitive detection of trace levels of bioconstituents. In addition to the demonstrated sensitivity permitted by the ZnO NRs-based platform, the approach has the advantages of rapid analysis, minimal volume requirements, and reusability. The multiplexed detection was achieved with 90 min of total assay time and only 60 μL of total bioreagent/sample volume using commonly employed fluorescence microscopy instrumentation. Further, the highly biocompatible ZnO NRs platform was found to withstand at least 25 repeated assays in complex biological and chemical reaction environments that include urine samples.

#### 5.2.4. Photonic Crystal-Enhanced Fluorescence

**Figure 10:**
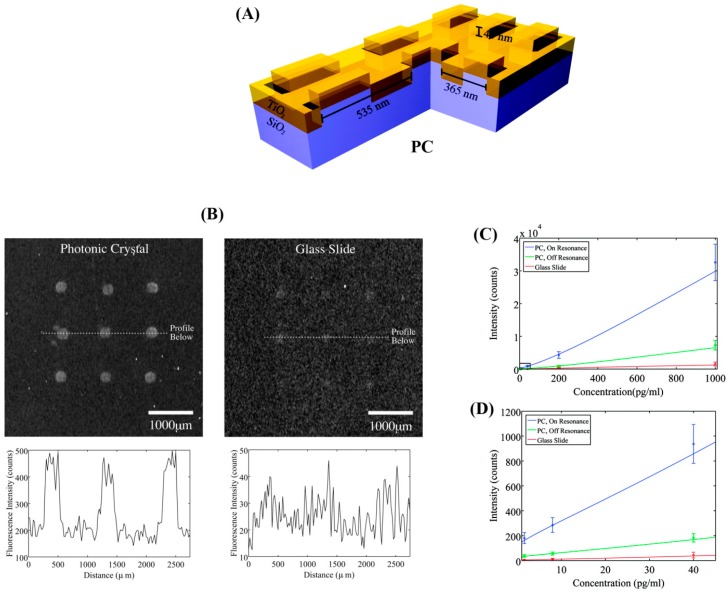
(**A**) The layout of the SiO_2_/TiO_2_ PC platform fabricated using a nanoreplica molding process; (**B**) Fluorescence data obtained from an identical immunoassay performed using a PC and a glass slide. The fluorescence images and the corresponding intensity profiles show that the SNR of the fluorescence assay is about 8 times higher than the ratio from the glass slide assay; (**C**) Net fluorescence intensity as a function of TNF-α concentration for the immunoassay performed on the PC on-resonance, the PC off-resonance, and the glass slide; (**D**) The lower concentration data in (**C**) are magnified to clearly display the results of the three lowest assay concentrations. Reproduced with permission from Ref. [91] Copyright (2008) American Chemical Society.

Photonic crystals (PCs) are periodic nanostructures of dielectric materials fabricated on a surface, whose photonic bandgaps are modulated to ban light propagation for specific wavelengths [89,90]. The ability of PCs to be tuned for a specific light wavelength to be monitored can be exploited in fluorescence detection. The detailed fundamental background for PCs is provided as a part of optical resonance sensors in Section 5.4. Although PCs may not be categorized as an optical resonator in a strict sense, they share common detection principles. The wavelength tuning capability of the PCs can be useful when immunoassay conditions suffer from autofluorescence from the assay platform, the medium, or any other fluorescing biological/chemical compounds present in the assay environment. The use of PCs in these cases may offer better signal collection and increase the signal-to-noise ratio (SNR) since the detection enables the monitoring of signals associated with target proteins only by tuning the detection wavelength to the emission of the fluorophore joined to the target proteins. The potential of PCs in enhancing fluorescence detection capabilities has been examined in cytokine immunoassays [91,92,93,94]. In one example shown in Figure 10, nanostructured PCs were fabricated using a nanoreplica molding process to obtain periodically modulated low and high refractive index (*n*) components of SiO_2_ (*n* of 1.45) and TiO_2_ (*n* of 2.35) layers [91]. Using this PC substrate, the detection SNR was increased by 5-fold for a sandwich assay of TNF-α using a Cyanine-5 (Cy5) label and a phosphate buffered saline (PBS) buffer. The sensor surface was illuminated with a collimated, polarized white light source and a UV-VIS spectrometer was used for collection of transmitted light. The PC-enhanced fluorescence assay of TNF-α yielded an improved DL of 6 pg/mL relative to a DL of ~18 pg/mL on a glass slide. 

#### 5.2.5. Modifications in Immunoassays

Other ways to improve the sensitivity of fluorescence detection and various improvement areas of traditional immunoassays have been addressed in recent efforts. Although broadly applied and not exclusive for cytokines, Table 3 shows three different examples of protein detection platforms created by various adjustments in the immunoassay steps. The single fluorescent molecule counting approach uses an additional elution step in a sandwich assay. After incubation to form the sandwich complex, the elution step is used to dissociate fluorescently labelled antibodies from the microbead complex in a concentrated volume. This concentrated volume of fluorescently labelled antibodies is then analyzed using flow cytometry. In the immuno-polymerase chain reaction (PCR) approach, the secondary antibodies in the sandwich immunoassay are conjugated with DNA labels whose strands are amplified exponentially in a downstream amplification process. The signal can then be quantified using a real-time quantitative PCR (qPCR) method. The single fluorescent molecule array scheme traps single microbeads in femtoliter (fL)-sized wells to remove background signals. Then, enzymatic amplification of the analyte binding events can be performed to a single microbead residing in each well to improve signal detection.

A signal amplification scheme for immunosensors based on hybridization chain reaction (HCR) schemes was recently demonstrated for cytokine measurements [95]. In this approach, a sandwich type of TNF-α antibody-antigen immunocomplex was incubated with streptavidin and a biotinylated initiator strand, which then induced the catalyzation of two complementary, kinetically trapped hairpins to self-assemble. The subsequent addition of SYBR Green I, a fluorescent molecule which intercalates in the grooves of DNA, allowed for quantitative detection of TNF-α by single molecule counting of each hybridization reaction. The sensor showed suitability using real serum, reporting a dynamic range of 1.28 to 25.6 pg/mL (i.e., 50 fM to 1 pM) for TNF-α with an assay time of longer than 12 h from sample introduction. The diffusion-controlled binding kinetics of traditional immunoassays were addressed by coupling a microsphere-based assay with a microfluidic device that created turbulent flow profiles from a U-shaped ‘serpentine’ geometry allowing for better sample mixing [96]. Coverage on the microsphere surface was achieved in seconds using this method which is a significant improvement in comparison to the 1–2 h incubation required for non-mixed samples, allowing near real-time fluorescence detection. A DL of 20 pg/mL for TNF-α was attained while using μL volumes with no washing steps.

Aptamer beacons have also been incorporated in cytokine detection employing fluorescence as the mode of signal transduction where DNA/RNA probes are selected from synthetic libraries [49,97,98,99,100]. These aptamer-based sensors exist in a variety of configurations which can include the release of quencher-labeled complementary strand or aptamer-bound fluorophore upon antigen binding as well as the use of graphene oxide (GO) or reduced grapheme oxide (RGO). In a Forster resonance energy transfer (FRET)-based approach, micropatterned aptamer beacons were utilized in the form of hybridized pairs of complementary strands, one labeled with a fluorophore and the other with a quencher. Upon displacement of the quencher strand by IFN-γ molecules, fluorescence signal was monitored as the quenching effects were reduced. This sensor scheme allowed a DL of 84.4 ng/mL (i.e., 5 nM) [97,98].

**Figure 11:**
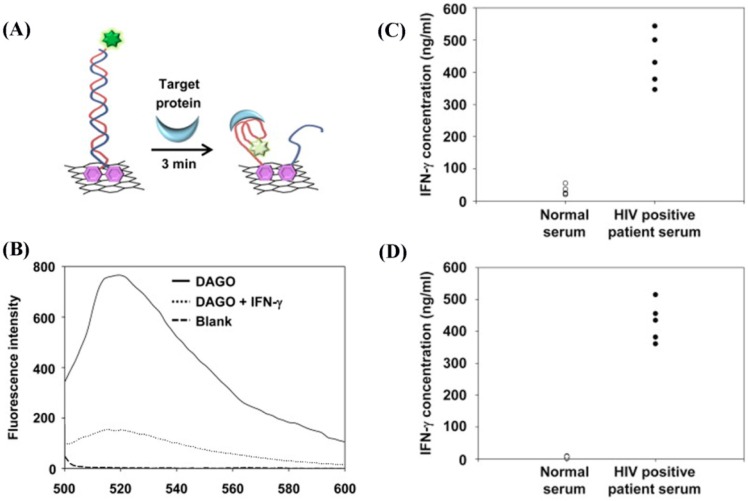
(**A**) Schematic illustration depicts the overall fluorescence assay for a target protein using double stranded aptamers on RGO (DAGO) nanosheets; (**B**) The fluorescence spectrum is obtained after incubating 1 μg/mL of IFN-γ with 10 μg/mL DAGO for 3 min; (**C**,**D**) IFN-γ assay results obtained by using DAGO (**C**) and an IFN-γ ELISA kit (**D**) are displayed. The assays were conducted using the same normal and HIV positive patient serum samples for comparison between the two methods. Reproduced with permission from Ref. [99] Copyright (2014) Elsevier.

Additionally, 2D nanomaterials have been used in conjunction with the FRET-based measurement scheme. GO [49] and RGO [99] have been employed to better anchor aptamers and fluorescence quenching was monitored upon IFN-γ binding. These strategies with GO and RGO achieved DLs of 25.3 fg/mL (1.5 fM) and 0.1 ng/mL (5.93 pM), respectively. Figure 11 shows the application of the RGO-based approach in IFN-γ detection. The sensing component anchored on a RGO substrate is composed of a target protein-specific aptamer decorated with a fluorescent probe of carboxyfluorescein (FAM) and a quencher of black hole quencher-1 (BHQ1), as shown in Figure 11A. When binding between the aptamer and protein occurs, the quencher is pulled closer to the fluorophore, decreasing the fluorescence intensity.

#### 5.2.6. Modifications in Fluorophores: QDs

**Figure 12:**
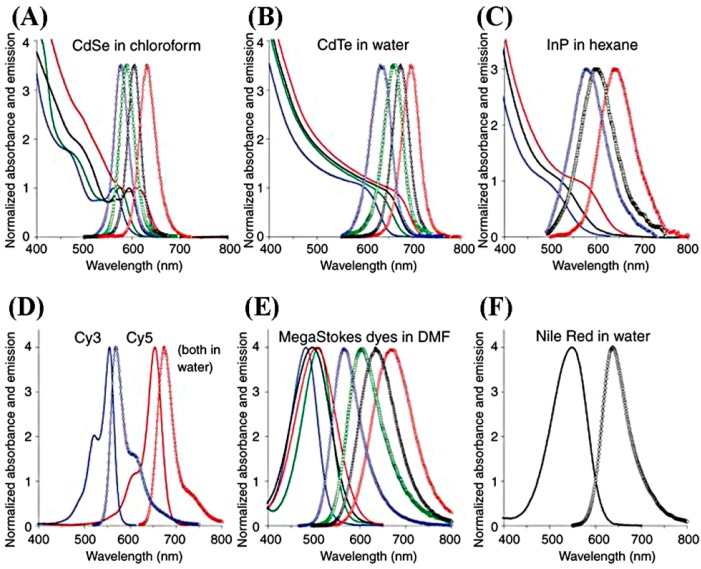
Comparison spectra of QDs and organic fluorescent dyes are displayed. Data shown as lines and symbols represent absorption and emission spectra, respectively. (**A**–**C**) QD spectra. Representative absorption and emission spectra are shown for the QDs of (**A**) CdSe, (**B**) CdTe, and (**C**) InP. (**D**–**F**) Organic dye spectra. Typical absorption and emission spectra for model organic dyes are displayed for (**D**) Cy3 and Cy5, (**E**) Megastokes and (**F**) Nile Red. Different colors in the spectra are coded by the size of the dye (blue < green < black < red). Reproduced with permission from Ref. [104] Copyright (2008) Nature Publishing Group.

Semiconducting NPs known as quantum dots (QDs) have been developed and used in fluorescence-based cytokine assays as alternatives for the traditional fluorescent dyes made from organic compounds. QDs represent a variety of inorganic compounds with core-shell structures. Examples include CdS/ZnS, CdSe/ZnS, CdSe/CdS, InAs/CdSe, ZnSe/CdSe, and CdTe/CdSe NPs. QDs are typically hydrophobic and, therefore, the core-shell complex is treated with polymeric or other hydrophilic coating layers in order to make them water soluble. The extended QD constructs also have chemical functionalization groups or biological moieties to equip them with the specificity needed in immunoassays. The final size of the QD complexes is 10–15 nm or even larger, and can reach the size range of a macromolecule [101]. Compared to traditional dyes, QDs can be excited with a broad band for absorption and, yet, their emission peaks are very narrow and symmetric, exhibiting large Stokes shift values of 300–400 nm. The absorption and emission spectra of several QDs are compared to traditional dyes in Figure 12. QDs can be fabricated to have absorption-emission wavelengths from near UV to near IR. Their quantum yield is high, while resisting photobleaching. QDs are also known to have longer lifetimes and higher photostabilities (~3 orders of magnitude larger) than organic dyes. All of these improvements by the use of QDs can lead to better fluorescence sensitivity in cytokine detection than traditional fluorophores [102,103,104]. Six different cytokines of TNF-α, IL-8, IL-6, MIP-1β, IL-13 and IL-1β were detected down to the pM concentration level in a study testing the ability of QD fluorophores to detect [101]. The assays were carried out in a Tris-buffer with a total of ~4 h of incubation time by using a QD probe of QD655.

### 5.3. Surface Plasmon Resonance

The aforementioned colorimetric and fluorescence techniques often require the use of labels such as biological tags (enzymes with chromogenic agents, native and modified green fluorescent proteins), fluorophores (organic dyes, semiconductor nanocrystals, and quantum dots), and other inorganic chemical reagents. In contrast, surface plasmon resonance (SPR) technique offers the benefit of label-free detection with good sensitivity [105,106,107,108,109] as well as time-resolved detection for single analytes [110,111]. Signals measured with SPR and localized SPR (LSPR) are both based on the small changes in the refractive index on the sensor surface upon analyte binding. For SPR sensors, momentum between surface plasmon and incoming light should be matched. LSPR sensors can present a tunable resonant frequency that does not necessitate momentum matching. Due to significantly enhanced local fields through confining the electromagnetic energy from the incident light in the nanoparticles, the sensitivities of LSPR sensors can be higher than those of SPR. Nonetheless, this effect is counterbalanced by the smaller plasmon-active adsorptive surfaces of the metal nanoparticles used in LSPR relative to the planar films in SPR.

#### 5.3.1. Fundamental Background for SPR and LSPR

A plasmon is a collective oscillation of free electrons in a metal. Under an external electric field, displacements of the collective electron oscillations occur with respect to the fixed ionic cores. The energy of these bulk plasmon oscillations at the plasma frequency is:

(5) $$E=\;\frac{h}{2\pi }\;\sqrt{\frac{Ne^{2}}{m\varepsilon_{0}}}$$

where *h* is the Planck constant, *N* is the electron density, *e* is the electron charge, *m* is the electron mass, and *ε*_0_ is the vacuum permittivity.

Plasmons occurring at the surface of a metal are referred to as surface plasmon polaritions (or simply surface plasmons). Light can be coupled into standing or propagating surface plasmon modes using a grating, a waveguide, or a prism. The propagating surface plasmon wave is a transverse magnetic (TM)-polarized wave with its magnetic vector oriented parallel to the plane of interface and perpendicular to the direction of propagation. Hence, p-polarized light is required to excite surface plasmons and the wavevector of incident light should match the wavevector of surface plasmons. The electric field of the surface plasmon wave is distributed in a highly asymmetric manner with the vast proportion (85%–90%) of the field centered in the dielectric. It has the maximum point at the metal-dielectric interface and decays evanescently into both the metal and dielectric layers. For the frequently used materials of Ag and Au, the surface plasmon wave propagating at the interface has a penetration depth of ~220 nm and ~160 nm, respectively, into the dielectric under 630 nm light, while both show ~30 nm penetration depths into the metal layer. The propagation constant of the surface plasmon wave in a semi-infinite system is given as [110]:

(6) $$k\sqrt{\frac{\varepsilon_{m}\;n_{d}^{2}}{\varepsilon_{m}+n_{d}^{2}\;}}$$

where *k* is the free space wavenumber, *ε_m_* is the dielectric constant of the metal, and *n_d_* is the refractive index of the dielectric. *ε_m_* is a complex number with its real and imaginary parts of *ε_mr_* + *iε_mi_*. Surface plasmon waves are supported by the system providing the condition of $\varepsilon_{mr}\;<-n_{d}^{2}$. For optical wavelengths, metals such as Au and Ag can meet this requirement. The surface plasmon wave propagates with high attenuation in the visible and near infrared spectral regions due to high losses in the metal. For 630 nm light, Au and Ag have 3 μm and 19 μm propagation length.

**Figure 13:**
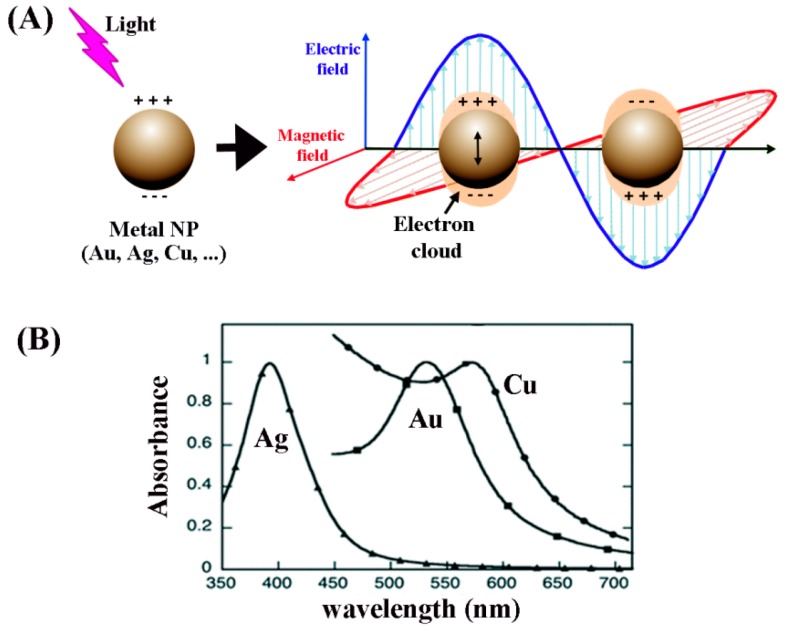
(**A**) An illustration of a localized surface plasmon around a metal nanoparticle; (**B**) Typical examples of surface plasmon peaks appearing in the visible range for the metals of Ag, Au, and Cu.

When surface plasmons are confined to a nanosized particle whose diameter is comparable to or smaller than the wavelength of light, as displayed in Figure 13, they are called localized surface plasmons and have important implications. For this localized surface plasmon, the electrical field near the nanoparticle is greatly enhanced and the enhancement is the largest at the nanoparticle surface while rapidly decreasing with distance. In addition, the optical extinction of the nanoparticle has a maximum at the plasmon resonant frequency whose extinction peak depends on the refractive index of the surrounding medium. The plasmon resonant frequencies for noble metals such as Au and Ag nanoparticles are in the visible wavelength range.

The extinction cross-section for nanoparticle plasmon resonance can be described as [112],

(7) $$\frac{18\pi \varepsilon_{m}^{3/2}V}{\lambda }\;\frac{\varepsilon_{2\;}\left(\lambda \right)}{{\left[\varepsilon_{1}\left(\lambda \right)+2\varepsilon_{m}\right]}^{2}+\varepsilon_{2}{\left(\lambda \right)}^{2}}$$

where *V* is the nanoparticle volume, *λ* is the wavelength of light, and *ε*_1_ (and *ε*_2_) are the real (and imaginary) dielectric constants of the dielectric medium at *λ*. When *ε*_1_ = −2*ε_m_*, the denominator in the above equation is minimized, leading to a maximized extinction cross-section. This explains the dependence of the LSPR extinction peak on the surrounding medium. For example, the expected wavelength where *ε*_1_ = −2*ε_m_* is about 520 nm for a AuNP (*ε_m_* = 1.7) in water.

**Figure 14:**
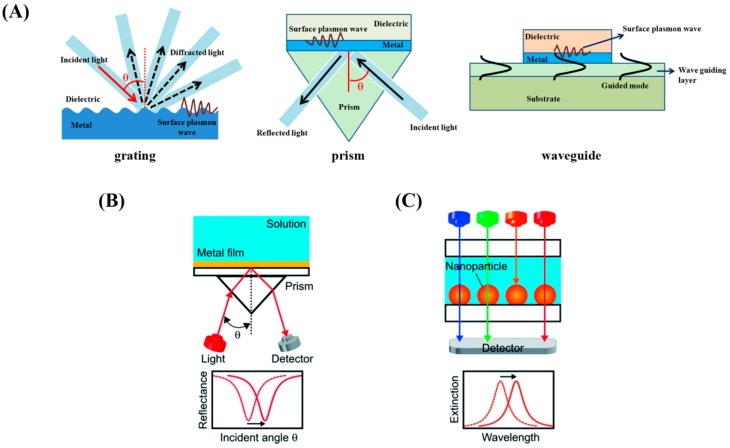
(**A**) The schematics display grating-, prism-, waveguide-based light coupling configurations of SPR. Reproduced with permission from Ref. [113] Copyright (2016) MDPI; (**B**,**C**) The illustrations show (**B**) a prism-based SPR sensor and its signal typically measured in terms of the change in the resonance angle and (**C**) a LSPR sensor detecting shifts in a spectral peak. Reproduced with permission from Ref. [114] Copyright (2014) Royal Society of Chemistry.

Traditional SPR sensors are configured to excite surface plasmon polaritons on the back of a metallic thin film (typically a Au film) by coupling polarized light with a prism at a resonant angle. The three common types of SPR biosensors are displayed in Figure 14A. The sensor signals are derived from the changes in the resonance angle of the light coupling condition [113] upon analyte binding to the sensor surface as shown in Figure 14B. Hence, detection of protein adsorption events on SPR sensor surface can provide data on biomolecular surface coverage, kinetics, and binding properties. Compared to the relatively inflexible arrangements required for momentum matching in SPR, LSPR works in a simpler configuration as shown in Figure 14C. The measured signal from LSPR is often based on the shift in the spectral peak due to the changes in the maximum extinction condition [114] following analyte binding.

#### 5.3.2. SPR and LSPR Applications in Cytokine Detection

SPR has been successfully exploited in cytokine analysis. To realize this, however, it was crucial to eliminate nonspecific binding of biomolecules from the bulk solution that do not directly participate in the assays. Without this improvement, the use of SPR with samples in unpurified, undiluted biological fluids often yielded relatively low sensitivity due to nonspecific binding signals and a temperature dependence when compared with commercially available assays [112]. Although SPR can operate label-free, SPR cannot be effectively applied to measure low concentrations of proteins with low molecular weights since the present applications require greater than 1 pg/mm^2^ of protein coverage [115]. It was later found that the use of mass-increasing tags can improve the SPR detection sensitivity. Therefore, recent advances of SPR-based cytokine and chemokine detection platforms cover improvements such as interfacing antibiofouling layers on the metal surface and labelling with mass-increasing tags.

A biofouling-resistant Au SPR sensor was developed by applying a coating of *N*-hydroxy-succinimide ester of 16-mercaptohexadecanoic acid (NHS-MHA) as an antibody support, which led to DLs of ~1 ng/mL for IL-1, IL-6, and TNF-α in a spiked cell culture medium [116]. A different type of antibiofouling layer involving a self-assembled monolayer of mercaptoundecanoic acid (MUA) and mercaptohexanol (MCH) was demonstrated on a commercial SPR sensor chip to achieve a similar DL of 1.3 ng/mL for IL-6 in a cell culture medium using direct antibody immobilization [117]. Another study has demonstrated a further improvement in detection sensitivity by introducing a number of changes in the assays. While using the MUA coating for antibiofouling, the mass of the target analyte was increased using AuNPs as well as secondary antibodies, which achieved a DL of 54.9 pg/mL for TNF-α in spiked human urine [118]. Beyond these strategies such as novel antibiofouling coatings and the addition of tags for additional mass to improve sensitivity, novel scaffolds derived from albumin binding domains (ABD) of protein G were also engineered for the detection of IFN-γ as superior biorecognition elements relative to primary capture antibodies [119]. Via covalent functionalization of a SPR sensor surface with the ABD scaffolds, a DL of 3.38 ng/mL (0.2 nM) was attained for IFN-γ in 2% depleted human blood plasma [120]. Another improvement approach includes sample purification ahead of SPR measurement. This method was used to detect IFN-γ released from CD4 T-cells in human blood by purifying the sample using a flow chamber coated with anti-CD4 antibodies for 10 min and subsequently measuring the secreted IFN-γ using an upstream SPR sensor coated with capture antibodies [19].

Though DLs have improved with the aforementioned advancements, SPR has seen limited translation into clinical settings whose tests often need to be carried out in complex, unpurified, undiluted biological fluids. Although a limited number of efforts has been made to develop multichannel SPR sensors [111], the technique cannot efficiently handle a large number of complex protein samples simultaneously in a rapid manner. The need for advanced optical instrumentation in SPR also becomes as a drawback when it comes to interfacing a wide variety of sample types and increasing its multiplexing capability. In order to overcome these difficulties, more flexible LSPR sensors and imaging-based SPR detection schemes have emerged as promising alternatives for clinical samples. 

**Figure 15:**
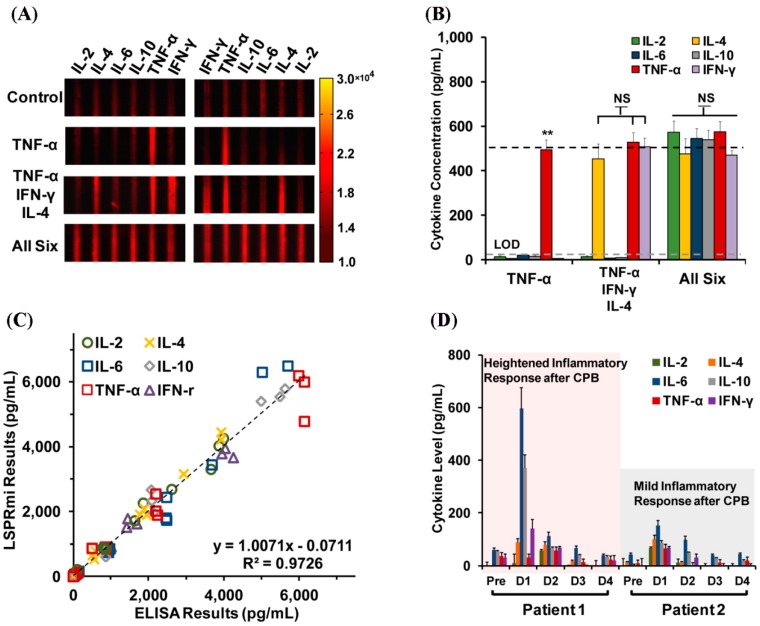
LSPR-based cytokine detection in healthy versus patient serum samples. (**A**) Dark-field images of AuNR microarrays within a single microfluidic detection channel loaded with different sample mixtures of recombinant cytokines (500 pg/mL for each cytokine) spiked in a serum matrix; (**B**) Cytokine concentrations are quantified for the samples in (**A**). The dashed black line represents the predetermined value of 500 pg/mL. The dashed grey line represents the DL of the LSPR microarray; (**C**) Correlations are made between the data obtained from the LSPR microarray assay and the gold standard ELISA for the spiked-in serum samples; (**D**) Five-day cytokine concentration variations were measured by the LSPR microarray assay for serum samples extracted from two post-cardiopulmonary bypass (CPB)-surgery pediatric patients. Reproduced with permission from Ref. [41] Copyright (2015) American Chemical Society.

A LSPR biosensor made of an ensemble of micropatterned, antibody-decorated AuNRs was demonstrated for the parallel detection of six cytokines using 1 μL of serum sample via dark-field imaging [41], as shown in Figure 15. Instead of measuring the wavelength change associated with analyte binding, the new approach was designed to scan the scattered light intensity across the microarrays of AuNR ensembles at a characteristic LSPR frequency using dark-field imaging optics. With a total assay time of 40 min, the LSPR sensor showed DLs of 20.56 (IL-2), 4.60 (IL-4), 11.29 (IL-6), 10.97 (IL-10), 6.46 (IFN-γ), and 11.43 pg/mL (TNF-α) with a dynamic range spanning from 10 pg/mL to 10 ng/mL. To demonstrate clinical integration, the variable cytokine expression profiles of two neonates were measured at multiple time-points after cardiopulmonary bypass surgery. The operation-induced inflammatory response producing elevated levels of IL-6 and IL-10 was then reported [41].

In addition to AuNRs, AgNPs have served as another metal commonly used in LSPR. A single molecule nanoparticle optical biosensor (SM-NOBS) consisting of AgNPs (~2.6 nm in diameter) conjugated with single antibody molecules was able to track real-time binding affinities and kinetics of TNF-α [121]. The AgNPs SM-NOBS allowed for the selective detection of TNF-α in the dynamic range of 1–200 ng/mL, but an assay time of 30–120 min was needed in the detection to reach the binding equilibrium.

**Figure 16:**
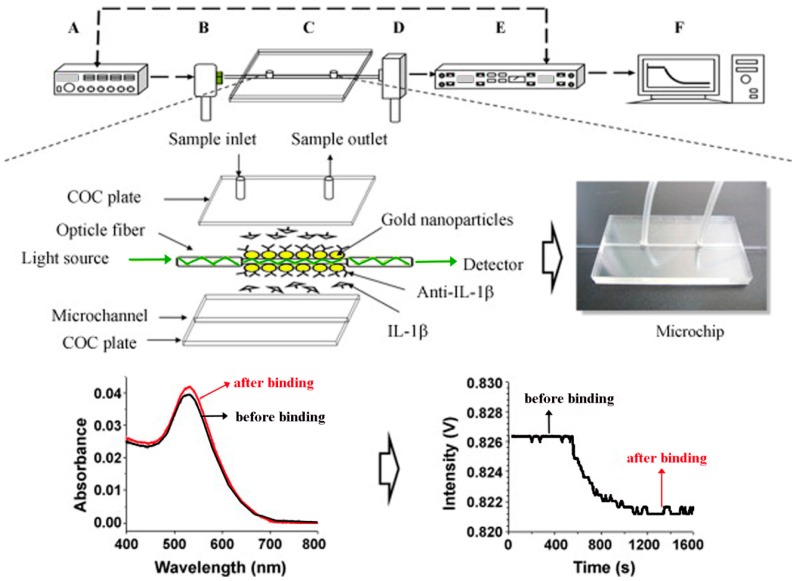
The schematics represent a FOPPR sensor setup and its working principle. The setup consists of a function generator, a light-emitting diode, a FOPPR sensing chip, a photodiode, a lock-in amplifier, and a computer, as indicated in A through F, respectively. Biomolecular binding at the surface of the functionalized AuNP layer (indicated in yellow) results in increased absorbance and decreased light intensity exiting the optical fiber (shown in green). Reproduced with permission from Ref. [122] Copyright (2010) Elsevier.

To improve the intrinsically low absorbance existing in NP submonolayers, a fiber-optic particle plasmon resonance (FOPPR) sensor was used in several studies [122,123] In the FOPPR sensor displayed in Figure 16, evanescent waves propagating along an optical fiber can excite AuNPs immobilized on the fiber surface. Similar to the conventional LSPR, the plasmon absorbance of antibody-functionalized AuNPs increases upon antigen binding, leading to a decrease in the light transmitted through the optical fiber. The signal can be also monitored in real time. The FOPPR device demonstrated a 21 pg/mL DL for IL-1β in synovial fluid with a linear dynamic range of 0.05–10 ng/mL which was comparable to the results using an ELISA kit, 22 pg/mL DL and 0.032–0.25 ng/mL range of linearity [122]. Unlike the relatively long assay time needed for LSPR, the FOPPR sensor was shown to display steady signal in less than 10 min. For TNF-α in synovial fluid, the FOPPR sensor yielded a 8.2 pg/mL (0.48 pM) DL although the detection required a relatively large sample volume of 200 μL and dilutions of the biological fluids to prevent bulk refractive index changes [123]. The dilution of samples is not just imposed on the SPR- and LSPR-based sensors but is often required for all optical detection modes relying on detecting the refractive index variation upon analyte binding. Refractive index changes can be triggered not only from analyte binding but also from fluctuating refractive indices associated with a complex biological environment. Hence, the dilution of real samples is required to avoid any false positives from refractive index changes of the sample media and this can lengthen the assay procedure.

### 5.4. Optical Resonance

**Figure 17:**
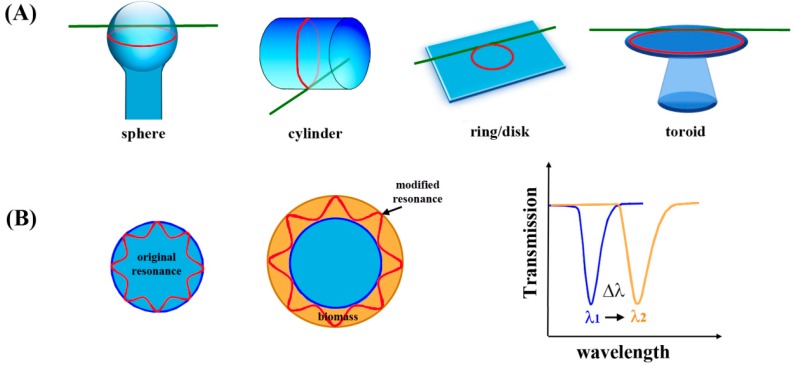
(**A**) Various geometries of microcavity resonators; (**B**) The original and modified optical waves in resonance are shown for the cases of before and after the binding of a bioanalyte on the sensor surface. The resonance wavelength change associated with this event is recorded in the transmission spectrum as Δ*λ*.

Microcavity resonators and microresonators, such as microtoroids, microspheres, and microrings, offer a label-free, real time approach for optical biosensing by monitoring changes in the local refractive index upon analyte binding [124,125,126,127]. These sensors, which are sometimes referred to as whispering gallery mode (WGM) resonators, can take various geometries as shown in Figure 17A. At a resonant wavelength, light that is coupled to the microcavity structure loses transmission intensity as it couples to the optical modes of the sensor material. Shifts in the resonant wavelength can be correlated with antigen binding events at the sensor surface. A body of work reports on the application of silicon-on-insulator (SOI) microring optical resonators for biodetection and promotes compatibility with existing semiconductor fabrication technologies [128,129,130,131]. More recently, SOI microring resonators based on sub-wavelength grating waveguides for use with TE polarized light have shown to improve detection sensitivity [132].

#### 5.4.1. Fundamental Background for Optical Resonance

Optical resonance occurs when an optical wave completes a revolution of the circumference of a resonator material and returns in phase by having an integer number of waves in one circumnavigation. When this condition happens, light collected reveals a dip at the resonance wavelength, *λ_res_*, in the transmission spectrum, as shown in Figure 17B. The binding of analyte molecules can increase the optical path length, perturbing the original resonance of the blank microcavity resonator. The shift in the wavelength of the light to compensate for this perturbation and return to the resonant condition is reflected in Δ*λ* of the resonance wavelength in Figure 17B.

#### 5.4.2. Related Background for Photonic Crystals

**Figure 18:**
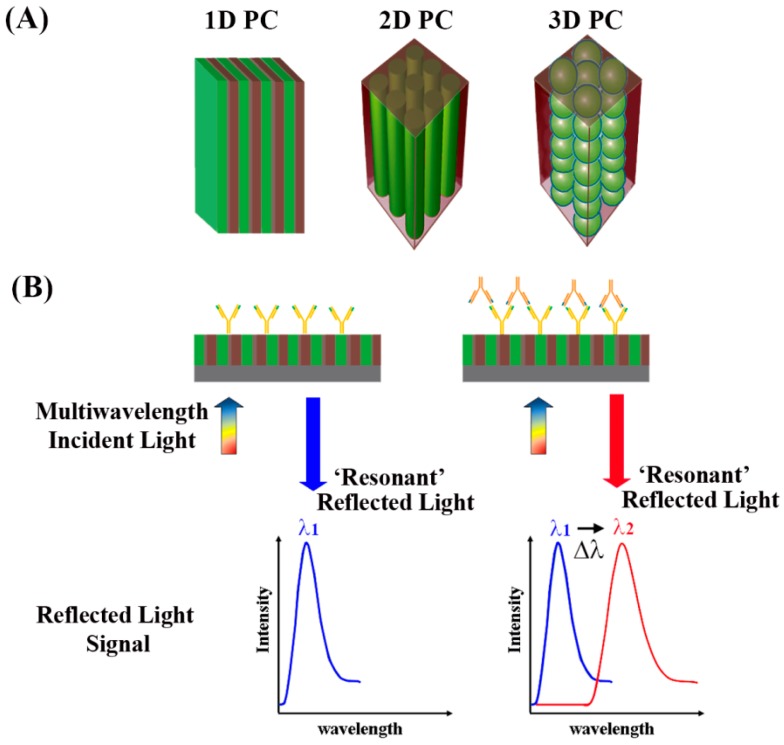
(**A**) 1D, 2D, and 3D PC systems are shown with the different colors corresponding to materials of different refractive indices; (**B**) Upon analyte binding, the resonant wavelength of the reflected light changes its wavelength from the original value by Δ*λ*.

As discussed earlier, PCs contain periodic nanostructures of dielectric materials fabricated on a surface, whose photonic bandgaps are modulated to ban light propagation for specific wavelengths [89,90]. Figure 18A shows the spatial arrangements of the dielectric materials constituting the three common types of 1D, 2D, and 3D PCs. When light is incident on the PCs normal to the surface, a certain wavelength of the incident light reflects according to the Bragg condition [133]:

*mλ* = 2*n_eff_d* (8)

where *m* is the reflective order, *λ* is the wavelength of the reflected light, *n_eff_* is the effective refractive index of the periodic structure, and *d* is the lattice period of the PC crystal in the direction of light propagation. When this diffraction condition is met, a high reflection is expected for *λ* and this wavelength is referred to as the resonant wavelength. Hence, the period and the effective refractive index of the PC can be tuned to adjust for different light wavelengths to be reflected. When biomolecules are adsorbed onto the PC surface, the surface-attached biomolecules lead to refractive index changes on the sensor surface. This leads to changes in the wavelength positioned at the center of the reflectance spectra, which is associated with the photonic stop band or photonic bandgap. This photonic bandgap wavelength responds very sensitively to any small change in the refractive index on the PC surface similar to the detection mechanisms for surface plasmon resonance in Section 5.3. Resulting changes in the peak wavelength reflected from the PC surface can be correlated with the biomolecular binding events as displayed in Figure 18B. Hence, the dielectric nanostructures on PC surfaces can serve as a sensitive, label-free biosensor platform [134]. Nanoscale structures also become important in PCs whose operation requires submicron periods to display the Bragg reflection condition in the visible range. The main advantage of PC-based sensors compared to the SPR types is that the properties of the optical surface waves can be changed by tuning the nanostructures in the PCs. In some reports, other benefits of PCs such as lower damping and lower loss than SPR have been also suggested [135,136,137].

PC surfaces have typically been produced by using highly specialized fabrication techniques such as electron-beam or nanoimprint lithography which are expensive and time-consuming. However, recent developments in high-throughput and polymer-based manufacturing processes have led to the commercial introduction of single-use PC sensors manufactured in a roll-to-roll fashion. For example, a new processing method of ‘horizontal dipping’ allowed for the fabrication of a PC-related sensor called a distributed feedback laser biosensor (DFBLB). The DFBLB was more compatible with conventional roll-to-roll manufacturing and could be incorporated into standard format microplates for cytokine detection [138]. It also promoted light confinement and amplification for a high Q factor (*λ*_0_/Δ*λ*_0_ of 1 × 10^4^ ~ 2 × 10^5^) without any reduction in wavelength sensitivity. However, the detection required a precisely tunable wavelength excitation source such as a frequency-doubled, Q-switched, yittrium aluminum garnet (Nd:YAG) pulsed laser. This was due to a high stringency needed for satisfying the light coupling condition to the DFBLB sensor, unlike the previous application of PCs.

#### 5.4.3. Optical Resonator Applications in Cytokine Detection

**Figure 19:**
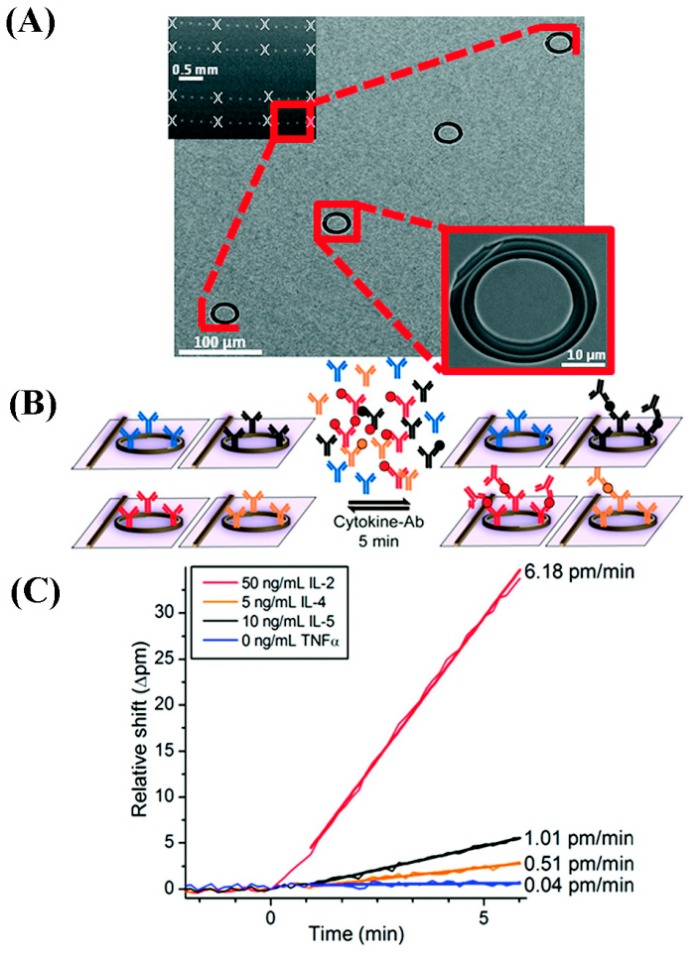
(**A**) Scanning electron microscopy images of a silicon microring resonator chip are shown. The chip consisted of an array of 30 μm-sized rings from which 32 rings were used for simultaneous signal monitoring. Each of the 30 μm ring resonator was accessed by a separate linear waveguide and interfaced with microfluidic channels for sample delivery; (**B**) The microrings were functionalized with capture antibodies specific for various cytokine targets. Following incubation of samples with a cocktail of secondary antibodies, the one-step sandwich immunoassay was monitored in real time for each ring. Cytokines bind specifically to their capture antibodies in a complex with the cognate detection antibody, thus enhancing the signal; (**C**) Multiple cytokines at varying concentrations were simultaneously quantified based on the initial slope (Δpm/min) of the sensor response upon sample introduction. The plot displays data from one representative ring for each cytokine assessed. Reproduced with permission from Ref. [140] Copyright (2011) American Chemical Society.

A silicon photonic microring resonator was created for temporal monitoring of IL-2 secretion from Jurkat T lymphocytes, a model cancer cell line used for studying T-cell activation. A detection scheme on the Si photonic microring resonator yielded a DL of 100 pg/mL [139]. After the addition of a secondary antibody serving as a ~150 kDa mass label to the relatively small IL-2 molecule of ~17.6 kDa, the magnitude of the shift in the resonant wavelength was enhanced from 15 to 40 pm due to the increased changes in the local refractive index upon adding the mass tag. The capture antibody was also able to be regenerated 20–30 times allowing for reusability of the single chip sensor. The detection platform was further improved by decreasing the total assay time from 45 min to 5 min for the parallel detection of IL-2, IL-4, IL-5, and TNF-α in serum-containing cell media. DLs ranging from 1.23 ng/mL to 2.1 ng/mL (68 to 119 pM) were achieved bu using a one-step sandwich assay on 32-element arrays of silicon photonic microring resonators [140]. Figure 19 displays the 32-element microring resonator sensors used in the cytokine detection. The resonant wavelength shift is sensitive to changes in the local refractive index near the sensor surface due to the high refractive index contrast between the waveguide and aqueous environment, providing a 63 nm confinement of the exponentially decaying evanescent field [141]. Under mass-transport limited conditions as is usually the case for trace biomolecular analyses, the initial slope of the signal response (shifts in the resonant wavelength as a function of time, Δpm/min) is dependent on analyte concentration according to Fick’s first law [124,140]. The rapid response in the aforementioned study was enabled by using an initial slope-based analysis method which has been shown to provide improved precision relative to equilibrium-based measurements since the former uses multiple data points in a range of time as opposed to the latter’s single time point response [124].

### 5.5. Optical Waveguides

#### 5.5.1. Fundamental Background for Optical Waveguides

Waveguides are dielectric structures which can confine electromagnetic waves and guide them through the optical medium via total internal reflection. Materials used for waveguides, therefore, have sufficiently high refractive indices compared to those of the bioassay solution and air and have appropriate dimensions to guide light of a particular wavelength. In telecommunications and optical interconnects, where waveguides were first developed, materials such as InGaAsP/InP, AlGaAs/GaAs, glass, LiNbO_3_, polymers, and silicon-on-insulator (SOI) have been employed to construct optical waveguides. In particular, Si-based waveguides, which can be produced in large scale via fabrication processes that are standard CMOS (complementary metal-oxide-semiconductor) technology compatible, serve as the most common waveguide material found in optical resonators and fiber optics. Optical fibers represent one of the most common waveguide forms and their uses in biosensing have been demonstrated as evanescent biosensors, waveguide biosensors, SPR- or surface enhanced Raman scattering (SERS)-based biosensors, fluorescence biosensors, and optrode biosensors. They typically utilize a sensing layer composed of biorecognition elements and dyes to generate signals which are attached to the fiber end.

**Figure 20:**
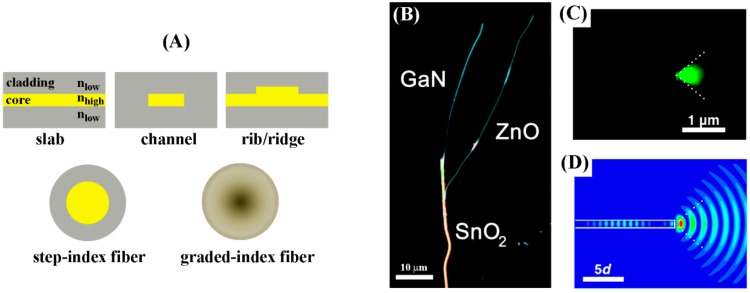
(**A**) Various structures of macroscopic and bulk waveguides are depicted; (**B**–**D**) 1D nanomaterial-based, subwavelength waveguides are displayed. (**B**) The waveguides are fabricated from using the optical cavities of a GaN NW, SnO_2_ nanoribbon, and ZnO NW with diameters between 130–250 nm. Reproduced with permission from Ref. [142] Copyright (2005) Proceedings of the National Academy of Sciences USA; (**C**,**D**) The conical emission of a low-order guided mode from a ZnO NR is shown in (**C**). The emission angle is approximately 90° (dotted lines). The image result in (**D**) is from a 2D finite difference time domain (FDTD) calculation of the square of the electric field of a light pulse emitted from a ZnO NR of diameter *d*. Reproduced with permission from Ref. [143] Copyright (2007) American Chemical Society.

Waveguides can be in planar and fiber forms of either macroscopic or nanoscopic dimensions. When the waveguides’ dimensions are smaller than the wavelength of guiding light, they are referred to as subwavelength waveguides. Optical waveguide structures are essential to couple and transmit light into biosensors. Therefore, in the aforementioned techniques such as SPR, PC, optical resonator, and fiber optic-based sensors, efficient light introduction to the active regions of the sensors often relies on the use of a macroscopic scale (functional dimension larger than the wavelength of light, >1 μm) waveguide. As for effective subwavelength waveguiding media, the application of individual ZnO NRs discussed earlier in biomolecular fluorescence enhancement is an example of passive waveguides. ZnO NRs were used for enhanced detection of the emitted optical signals from biomolecules by guiding the light in the visible wavelength range through the NR that had a width smaller than the wavelength of the light. Figure 20 displays both macroscopic and nanoscale optical waveguides which utilize the optical cavities of slab, channel, rib/ridge, fiber, NW, NR, and nanoribbon.

In a waveguide, the propagation of the light depends on its wavelength. The optical waveguide has a maximum wavelength of light above which the light starts to attenuate rather than propagate. A light wave repeating after two reflections is called an eigenmode or mode of waveguide. Depending on the physical dimensions of the waveguides and the light wavelength to be guided, waveguides can be classified as a single-mode or a multimode medium. In general, when the active dimension of the waveguide is small (or thick), only one light mode (or multiple modes) can be guided. For example, for the 1D nanomaterial-based subwavelength waveguides, the single-mode cutoff diameters in air are determined as approximately 140 nm (*λ* = 365 nm) or 265 nm (*λ* = 600 nm) for SnO_2_ nanoribbons, 112 nm (*λ* = 365 nm) for GaN NWs, and 140 nm (*λ* = 380 nm) or 220 nm (*λ* = 510 nm) for ZnO NW [142]. An equation developed in fiber optics [144,145] was used to estimate the diameter range of the nanostructures below which light can be effectively guided, particularly by considering the *V* value of 2.405 for single mode guiding by a cylindrical waveguide [144,145].

(9) $$\lambda =\pi \;d_{cutoff}\;{\left(n_{NR}^{2}-\;n_{air}^{2}\right)}^{1/2}/V$$

*λ*, *d_cutoff_*, *n_NR_*, and *n_air_* are the wavelength of light, cutoff diameter for single mode guiding, the refractive index of the nanomaterial waveguide, and refractive index of air, respectively. *V* is the normalized frequency parameter of the waveguide.

The ratio between the integrated electromagnetic field intensity axially guided inside the nanomaterial cavity to the transversely diffracted light can be used for estimating the degree of light coupling and guiding axially along a 1D nanomaterial waveguide, regardless of the types of carried modes. This ratio is known as the fractional mode power (*η*) expressed in terms of *V* [144,146].

(10) $$\eta =1-(2.405\exp\left(-\frac{1}{V}\right){)}^{2}\;V^{-3}$$

The above assessment for a cylindrical waveguide indicates that thin NWs are not suitable for maximizing light guiding since light cannot be effectively confined within the active medium due to diffraction. As an example, for *λ* = 500 nm light travelling in a ZnO NR, greater than 80% of the electromagnetic field intensity is expected to be retained within the NR for the lowest-order guided-mode if the NR diameters are larger than 200 nm. On the contrary, less than 30% of the intensity is present in the NR for a ZnO NR less than 100 nm in diameter. Hence, for biosensor applications, the physical dimensions of the waveguides, such as the widths of 1D nanomaterials, should be carefully tuned to best couple and guide the light of specific wavelength and desired modes.

#### 5.5.2. Waveguide Applications in Cytokine Detection

The utilities of different waveguides are found to be useful in many optical detection areas and, often, their uses are combined with other optical sensing modes rather than functioning as a sole detection mechanism. Therefore, specific examples of the waveguide applications in cytokine detection are discussed throughout this Review. For example, macroscopic waveguides were used to guide and propagate incident light into the sensor channels of SPR, optical fibers, PCs, and optical resonators. In another example, nanoscopic waveguides were exploited in coupling and subwavelength waveguiding of the fluorescence emission upon cytokine binding. In particular, evanescent waves are an important part of the waveguide structures employed for biosensing [147,148]. Specifically, evanescent waves in waveguide structures are used to probe bioanalytes or to enhance optical signal via evanescence coupling in many optical biosensor devices. These aspects will be discussed in detail in the following section.

### 5.6. Surface Evanescence

#### 5.6.1. Fundamental Background for Evanescent Waves

Light launched into a waveguide that is placed into a dielectric medium of a lower refractive index (*n_medium_*) confines all the light which enters with an angle of incidence greater than the critical angle (*θ_c_*) inside the waveguide based on total internal reflection as shown below:

(11) $$\theta_{c}=\;\sin^{-1}\left(\frac{n_{medium}}{n_{waveguide}}\right)$$

**Figure 21:**
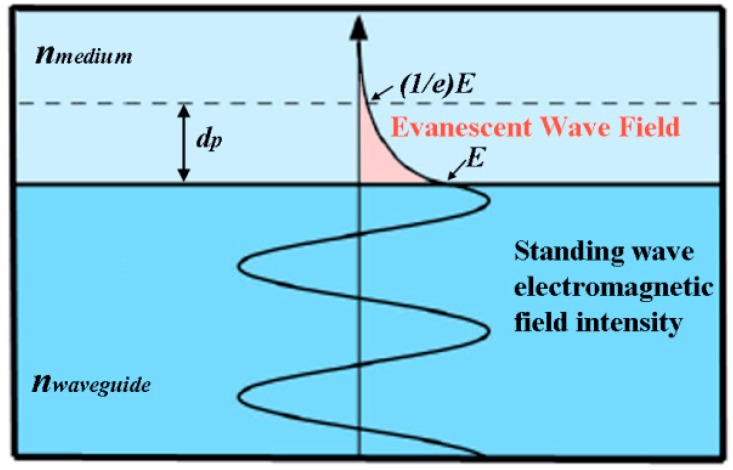
Illustration of a standing wave pattern formed near the interface between a waveguide with a higher refractive index and a medium with a lower refractive index (*n_waveguide_* > *n_medium_*) and the exponentially decaying evanescent wave with the penetration depth of *d_p_*.

Under this condition, the Fresnel transmission coefficients for the transverse electric (TE) and transverse magnetic (TM) waves are non-zero and an electromagnetic field extends out from the waveguide/medium interface into the lower refractive index medium [149]. This is known as an evanescent wave and decays exponentially with the distance of *d* into the medium, as illustrated in Figure 21.

The penetration depth (the distance at which the electromagnetic field strength is 1/*e* of its value at the interface) can be approximated by the equation given below and it extends approximately up to ~100 nm away from the interface [147]:

(12) $$d_{p\;}=\frac{\lambda }{2\pi }\;{\left[{\left(n_{waveguide}\right)}^{2\;}\sin^{2}\theta -{\left(n_{medium}\right)}^{2\;}\right]}^{1/2}$$

*d_p_* is the penetration depth of the evanescent field, *λ* is the light wavelength, and *θ* is the incident angle. This exponential decay of the electromagnetic field confines read-outs of any optical signals to within a certain distance from the waveguide surface, making the optical interference or background contribution from other components in the medium negligible. This high sensitivity to the immediate environment next to the waveguide surface has been exploited in a number of optical biosensors, including SPR, interferometry, fiber optics, and fluorescence. One of the critical aspects in an evanescent wave-based biosensor is to optimize the power available for excitation. For evenly distributed excitation, the fraction of power in the evanescent wave is given as [147],

(13) $$\frac{P_{0}}{P}=\;\frac{\nu }{N{\left(2N-2\nu \right)}^{1/2}}$$

(14) $$N=\;\frac{V^{2}}{2}$$

(15) $$V\;=\;\frac{\pi D}{\lambda }\sqrt{n_{waveguide}^{2}\;-\;n_{medium}^{2}}$$

where *P*_0_ is the power carried in the evanescent wave, *P* is the total power of that mode, *N* is the approximate mode capacity of the fiber waveguide, *V* is the *V*-number as defined previously, *D* is the diameter of the fiber waveguide, and *ν* is the mode order.

In 1D nanomaterial-based subwavelength waveguides, the role of evanescent waves becomes even greater since a larger fraction of light is known to be guided as a surface evanescent wave. The increased fraction of light outside enhances the intensity of an evanescent field and its penetration depth into the surroundings. In theoretical simulations based on the MIT Photonic Bands (MPB) program and Finite Difference Method (FDM), the penetration depth of the evanescent field around a hexagonal ZnO NR with a diameter of 500 nm was predicted as approximately 125 nm when an excitation of 761 nm was used [150]. Hence, these simulation results on the evanescent field of a ZnO NR show a relatively large decay length into the surrounding, which can be exploited in biosensing.

#### 5.6.2. Evanescent Wave Applications in Cytokine Detection

**Figure 22:**
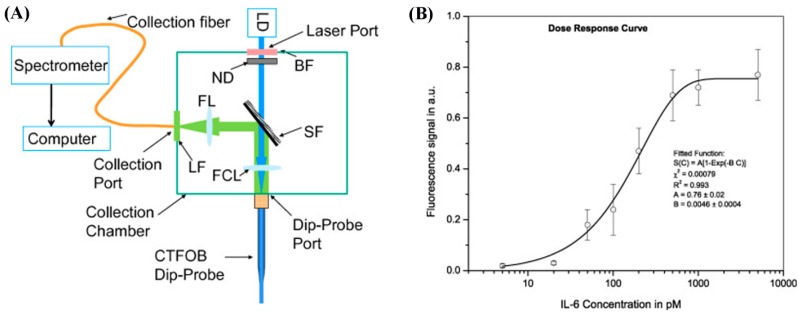
(**A**) A diagram of the CTFOB measurement setup is shown. LD, BF, ND, SF, FL, LF, and FCL denote the laser diode, band pass filter, neutral density filter, short pass filter, focusing lens, long pass filter, and focusing-collecting lens, respectively; (**B**) A dose-response curve is generated from the average fluorescence signal of various IL-6 concentrations. Reproduced with permission from Ref. [151] Copyright (2009) Elsevier.

An example of evanescent coupling of fluorescence emission is a combination tapered fiber-optic biosensor (CTFOB). Fiber-optic based biosensors can be operated using labeled or label-free techniques both of which take advantage of the evanescent waves propagating along the fiber creating enhanced electromagnetic fields at the sensor surface. However, auto-luminescence and nonspecific binding of uncomplexed, residual labeled antibodies can be a potential source of signal hampering and, therefore, these issues need to be effectively dealt with for cytokine detection. When used for IL-6 detection, the CTFOB dip-probe was able to achieve a DL down to 0.12 ng/mL (5 pM) after an appropriate blocking step to cut down nonspecific binding signal. In the sandwich assay scheme used, the dip-probe immobilized with capture antibody was first incubated in an IL-6 sample and subsequently in the detection antibody for 3 h. Fluorescence was then recorded using evanescent field excitation through the CTFOB, as shown in Figure 22. A strong reduction of the nonspecific binding signal was attained by mixing the labeled antibodies with a relatively high concentration of egg albumin (EA) molecules of more than 10^6^:1, which resulted in the competition of EA with the labeled antibodies contributing to nonspecific adsorption [151]. The serum IL-6 levels were further verified against a commercial bead-based multiplexed assay in a microplate format. The beads were labelled with assorted dyes at different levels for identification of multiple beads addressing different analytes via flow cytometry. Similar relative trends of IL-6 concentrations were reported despite the different absolute values, which were possibly due to the buffers changing the serum matrix for the bead-based assay. A DL of ~0.12 ng/mL and 100% specificity were determined for the CTFOB dip-probe based on a receiving operating characteristics (ROC) analysis [152].

### 5.7. Interferometry

**Figure 23:**
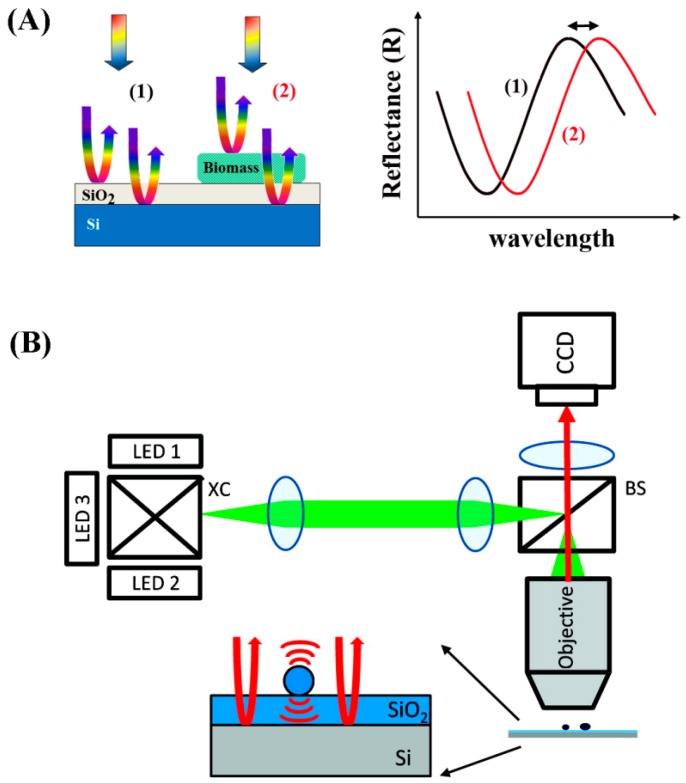
(**A**) Schematics of IRIS showing the interference of light reflected from the reference plane of the Si-SiO_2_ interface with the light from the top surface. Added biomass results in the wavelength-dependent reflectivity, as indicated with a double-headed arrow; (**B**) The overall layout for the optical setup in IRIS including a x-cube (XC) used to combine the beams of the different light emitting diodes (LEDs), a beam splitter (BS), and a charged coupled device (CCD) detector. Panel (**B**) is reproduced with permission from Ref. [154] Copyright (2010) American Chemical Society.

Broadly defined, interferometry refers to measurement techniques based on interferences of the light wave. In interferometry-based biosensing via an interferometric reflectance imaging sensor (IRIS), the detection is based on spectral reflectivity due to the increase in layer thickness following analyte accumulation on the sensor surface, as depicted in Figure 23. As a result, the optical path length changes due to the added mass before and after the bioanalyte binding. This in turn leads to a shift in the spectral reflectivity defined as *R*, as displayed in Figure 23 [153].

(16) $$R\;=\frac{r_{1}^{2}\;+\;r_{2}^{2}+\;2r_{1}r_{2}\cos2\phi }{1+\;r_{1}^{2}+\;r_{2}^{2}\;+\;2r_{1}r_{2}\cos2\phi }$$

(17) $$r_{1}=\frac{n_{2\;}-\;n_{1}}{n_{2}\;+\;n_{1}},\;r_{2}=\frac{n_{3}\;-\;n_{2}}{n_{3}\;+\;n_{2}},\;\phi \;=\;\frac{2\pi d}{\lambda }n_{2}\cos\theta$$

*r*_1_ and *r*_2_ are the Fresnel reflection coefficients and *n*_1_, *n*_2_, and *n*_3_ are the indices of refraction corresponding to air, SiO_2_, and Si, respectively. *λ* denotes the wavelength of incident light, *θ* is the angle of incidence, and *d* is the thickness of the bioanalyte.

The real-time detection of IL-6 in cell culture medium using an IRIS was recently reported [155]. The lowest achieved DL in the IRIS sensor was 2.7 ng/mL for IL-6. Although IRIS does not require any optical labels for signal detection, the reported DL was resultant of combining secondary antibodies for signal amplification before IL-6 deposition onto the sensor surface, similar to the mass tagging steps in SPR and optical resonance discussed above. 

## 6. Concluding Remarks and Outlook

This Review outlines existing and emerging cytokine sensors whose transduction mechanisms consist of optical detection modalities and whose configurations incorporate nanomaterials for signal enhancement. The fundamentals behind the key phenomena and principles serving as the bases of the promising optical biosensors are also discussed. They range from the long-established modes of colorimetry and fluorescence to the more recently developed modes of surface plasmon resonance, optical resonance, subwavelength waveguiding, evanescence coupling, photonic crystals, and interferometry. Specific attributes of 0D, 1D, and 2D forms of nanomaterials are brought together from the literature and their contributions to signal enhancement in different optical detection modes are explained in the context of cytokine detection. Nanomaterials discussed include Au and Ag NPs, semiconducting NPs (QDs), rare earth metal oxide NPs, Au thin films, ZnO NRs, SnO_2_ NWs, GO, and RGO. Examples of their applications in optical sensors are also featured specifically for cytokine detection and key achievements of the assorted nanomaterial-based optical sensors to date are summarized. 

The advancements made by the different optical sensors discussed in this Review have paved the way for the ultrasensitive quantification of multiple cytokine panels, presenting sensor capabilities that could not be attained by conventional optical detection means. It is expected that further advancements in emerging optical sensor technologies will continue to enable simpler and even more sensitive tests. Some of the new optical sensor platforms, such as the SPR sensors, have already been made into commercial products. Yet, the current implementation of even those commercially available technologies is largely in very limited areas of specialized research and analytical laboratories. This, in turn, makes them expensive and challenging to translate into clinical and hospital settings that monitor the general population.

For bench top-driven applications aiming to promote fundamental studies in basic science and clinical research laboratories, the emerging optical sensors have been successfully demonstrated as standalone platforms as well as in combination with traditional techniques. In these laboratory settings, various new optical sensors can serve effectively to enable stratification of new biomarkers and unambiguous detection of ultralow-expressed proteins, as evidenced by the wide range of optical detection platforms discussed in this Review. For these new biosensors to be even more useful and widely applicable to a variety of cytokine biomarkers, future endeavors should aim to deliver a complete evaluation of all important sensor characteristics listed in Section 3, instead of addressing only a subset of the criteria such as sensitivity and selectivity. 

Many challenges still lie ahead when evaluating the clinical utility of different optical sensors. To serve as an effective biomarker screening tool and clinical diagnostic guide, new sensor platforms should offer high detection sensitivity and a wide dynamic range suitable for screening real patient samples. In addition, the simultaneous analysis of multiple cytokines has become increasingly important. Sensors achieving the quantitative detection of cytokines while permitting the use of diverse types of samples with minimal processing are also essential for new sensor technologies, especially if their applications are geared for clinical and point-of-care environments. Hence, the potential impacts of novel optical sensor technologies will be the greatest when such multiplexed cytokine detection can be carried out from a variety of sample types without compromising the detection sensitivity and dynamic range. Particularly for the more recently developed optical techniques, additional sensor criteria such as ease of operation and user familiarity should be addressed to promote their widespread applicability in real world practices. For straightforward sensor operation and accurate signal interpretation by end users with minimum training, a fully packaged suite of hardware and software will also need to be developed to harness potential complexities in instrumental components and to handle sophisticated modelling and fitting routines involved with the measurements.

## Figures and Tables

**Table 1:** Various cytokines and their commonly used abbreviations. Each cytokine is listed along with its alternative name and the relevant section in the review.

Abbreviation (Molecular Weight) Alternative Designation	Cytokine Name	Section of Sensors Discussed	Cytokine Class
**EPO** (23.2 kDa)	Erythropoietin		Growth Factors (stimulate the proliferation and differentiation of cells)
**G-CSF** GM-DF, Granulocyte macrophage differentiation factor CSF3	Granulocyte colony stimulating factor		
**GM-CSF** CSF2	Granulocyte macrophage colony stimulating factor		
**M-CSF** CSF1	Macrophage colony stimulating factor		
**VEGF** (A-D) VPF, vascular permeability factor	Vascular endothelial growth factor	5.2.1 5.2.2	
**IFN-α** Leukocyte interferon	Interferon-α		Interferons (originally known related to their antiviral activity)
**IFN-β** Fibroblast interferon	Interferon-β		
**IFN-γ** (16.9 kDa) Immune interferon Type II interferon	Interferon-γ	5.2.2 5.2.5 5.3	
**IL-2** (17.6 kDa) T cell growth factor	Interleukin-2	5.1.1 5.3 5.4	Lymphokines (produced largely by lymphocytes)
**IL-3** Mast cell growth factor Persisting cell stimulating factor	Interleukin-3		
**IL-4** (14.9 kDa) BCGF-1, B cell growth factor-1 BSF-1, B cell stimulating factor-1	Interleukin-4	5.1.1 5.3 5.4	
**IL-5** (15.2 kDa) TRF, T cell replacing factor EDF, Eosinophil differentiation factor	Interleukin-5	5.4	
**IL-6** (23.7 kDa) IFN-β2, Interferon β-2 PGF, Plasmacytoma growth factor B cell differentiation factor CDF, Cytotoxic T cell differentiating factor	Interleukin-6	5.1.1 5.2.1 5.2.2 5.2.6 5.3 5.5 5.6 5.7	
**IL-9** T cell growth factor III	Interleukin-9		
IL-10 (20.5 kDa) CSIF, Cytokine synthesis inhibitory factor	Interleukin-10	5.1.1 5.2.1 5.3	
**TNF-β** Lymphotoxin alpha TNFSF1	Tumor necrosis factor-β		
**IL-1α** (30.6 kDa) Osteoclast activating factor Melanoma growth inhibitor factor Tumor inhibitory factor-2	Interleukin-1α	5.3	Monokines (predominantly produced by mononuclear phagocytes)
**IL-1β** (30.6 kDa) Same as IL-1α	Interleukin-1β	5.1.1 5.2.1 5.2.2 5.2.6 5.3	
**IL-12** CLMF, Cytotoxic T lymphocyte maturation factor NKSF, NK cell stimulatory factor	Interleukin-12		
**IL-15** IL-T, Interleukin-T	Interleukin-15		
**TNF-α** (25.6 kDa) TNFSF2 Cachectin	Tumor necrosis factor-α	5.1.1 5.2.3 5.2.4 5.2.5 5.2.6 5.3 5.4	
**IP-10** (10.9 kDa)	Interferon inducible protein-10	5.2.1	Chemokines (chemotactic, influencing cell migration and activation)
**IL-8** (11.1 kDa) CXCL8, C-X-C motif ligand 8 MDNCF, Monocyte derived neutrophil chemotactic factor	Interleukin-8	5.2.1 5.2.3 5.2.6	
**MCP-1/MCAF** (11.0 kDa) CCL2, C-C chemokine ligand 2	Monocyte chemotactic protein-1/monocyte chemotactic and activating factor	5.2.1	
**MIP-1α** CCL3, C-C chemokine ligand 3 LD-78 pAT464	Macrophage inflammatory protein-1α		
**MIP-1β** (10.2 kDa) CCL4, C-C chemokine ligand 4	Macrophage inflammatory protein-1β	5.2.1 5.2.6	
**RANTES** (9.9 kDa) CCL5, C-C chemokine ligand 5	Regulated upon activation, normal T-cell expressed and secreted	5.2.1	
**IL-7** Lymphopoietin-1	Interleukin-7		Others (biological action mainly observed with other cytokines)
**IL-11**	Interleukin-11		
**LIF** Differentiating stimulating factor	Leukemia inhibitory factor		
**TGF-β**	Transforming growth factor-β		

**Table 2:** Nanomaterials are classified according to their dimensionality along with their unique optical properties leading to signal enhancement in various cytokine sensors to be discussed in the ensuing sections.

	Shape	Nanomaterial Type	Unique Property for Signal Enhancement in Optical Sensors	Sensor Section Discussed
0D	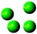 e.g., 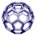	Nanoparticle Nanocage Bucky-ball (Fullerene) Quantum dot	Color enhancement Metal enhanced fluorescence Improved fluorophore properties Localized surface plasmon resonance	5.1.1 5.2.2 5.2.6 5.3 5.6
1D	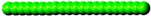 e.g., 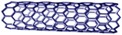	Nanotube Nanowire Nanorod Nanofiber Nanoribbon Quantum wire	Electromagnetic field concentration (lightning rod effect) Subwavelength waveguiding Evanescent waveguiding Light coupling and confinement	5.2.2 5.2.3 5.4 5.5 5.6
2D	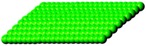 e.g., 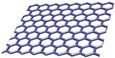	2D thin film 2D sheet Quantum well	Metal Enhanced Fluorescence Surface plasmon resonance	5.2.2 5.2.5 5.3 5.4

**Table 3:** Sensitivity improvements made by modifying immunoassay steps. * The numbers shown in the row can be considered as a general point of reference as they represent the best values found in various commercial products using the specified approach.

	Single Fluorescent Molecule Counting	ImmunoPCR qPCR	Single Fluorescent Molecule Arrays
Sensitivity level *	sub-pg/mL	fg/mL-pg/mL	fg/mL
Functional read-out	flow cytometry	real-time qPCR	Fluorophores/enzymes
Sample volume requirement per data point *	100 μL	30 μL	100 μL
Bead versus Plate	bead or plate	plate	bead
Signal amplification	No	exponential	enzymatic
Miniaturization	No	No	Yes
Label	Fluorophore	DNA tag	enzyme
Multiplexing capacity	No	2-plex	10-plex
Dynamic range *	6 logs	4 logs	4 logs
Throughput *	4 × 96-well plates/day	5 × 96-well plates/day	5 × 96-well plates/day
Sample replication	Duplicates	Duplicates	Duplicates
